# Quantum lattice dynamics and their importance in ternary superhydride clathrates

**DOI:** 10.1038/s42005-023-01413-8

**Published:** 2023-10-16

**Authors:** Roman Lucrezi, Eva Kogler, Simone Di Cataldo, Markus Aichhorn, Lilia Boeri, Christoph Heil

**Affiliations:** 1 Institute of Theoretical and Computational Physics, Graz University of Technology, NAWI Graz, 8010 Graz, Austria; 2 Dipartimento di Fisica, Sapienza Università di Roma, 00185 Rome, Italy; 3 Enrico Fermi Research Center, Via Panisperna 89 A, 00184 Rome, Italy

**Keywords:** Superconducting properties and materials, Computational methods

## Abstract

The quantum nature of the hydrogen lattice in superconducting hydrides can have crucial effects on the material’s properties. Taking a detailed look at the dynamic stability of the recently predicted BaSiH_8_ phase, we find that the inclusion of anharmonic quantum ionic effects leads to an increase in the critical dynamical pressure to 20 GPa as compared to 5 GPa within the harmonic approximation. We identify the change in the crystal structure due to quantum ionic effects to be the main driving force for this increase and demonstrate that this can already be understood at the harmonic level by considering zero-point energy corrections to the total electronic energy. In fact, the previously determined critical pressure of kinetic stability *p*_kin_ = 30 GPa still poses a stricter bound for the synthesizability of BaSiH_8_ and similar hydride materials than the dynamical stability and therefore constitutes a more rigorous and accurate estimate for the experimental realizability of these structures.

## Abstract (web summary)

The quantum nature of the hydrogen lattice in superconducting hydrides can have crucial effects on the material’s properties. In this work, the authors focus on the low-pressure superconductor BaSiH_8_ and identify the structural change due to quantum ionic effects to be the main driving force in increasing the critical pressure of dynamic stability.

## Introduction

The discovery of high-temperature superconductivity in H_3_S at extreme pressures^1^ stimulated an intense hunt for novel hydride compounds with even higher critical temperatures (*T*_c_), spearheaded by computational material discovery^2–6^. One prominent example is LaH_10_, which has been shown to superconduct up to temperatures of 265 K at pressures of ~190 GPa^7,8^.

While it is very tempting to continue searching for materials with record-breaking *T*_c_’s^9–11^, lowering the required stabilization pressures is even more important in view of technological applications^11–20^.

In a recent paper^15^ some of us proposed a strategy to bring the stabilization pressures of high-*T*_c_ hydrides closer to ambient pressure, based on the concept of an optimized *chemical precompression*. In fact, we identified LaBH_8_, a hydride superconductor with a *T*_c_ > 100 K, dynamically stable at an unprecedentedly low pressure. In a follow-up work, we showed that other hydride superconductors with the same $Fm\bar{3}m$ *XY*H_8_ structural template can be identified with even lower critical pressures of stability, such as SrSiH_8_ and BaSiH_8_^19^. The latter is particularly interesting, as it remains dynamically stable down to 3 GPa. We note in passing that these estimates of dynamical stability were based on anharmonic frozen-phonon calculations.

All of the mentioned $Fm\bar{3}m$ *XY*H_8_ compounds are thermodynamically stable only at pressures above ~100 GPa, and metastable below. A conceivable route to synthesize these materials would hence be to obtain them at high pressures where they are thermodynamically stable, and quench them to lower pressures. Such a procedure has recently been successfully employed to realize the $Fm\bar{3}m$ LaBeH_8_ at around 110 GPa with subsequent quenching down to a pressure of ~80 GPa^21^.

The standard criterion employed in literature to estimate how far a metastable phase can be quenched down in pressure is *dynamical* (phonon) stability. However, dynamical stability indicates only that a structure is in a local minimum of the potential energy surface. To estimate its actual lifetime (*kinetic* stability) one needs also to estimate the height of the barriers that separate the current minimum from other minima. In a previous work^19^, some of us introduced a rigorous method to assess the kinetic stability pressure *p*_kin_ by explicitly calculating the energy barrier protecting the metastable $Fm\bar{3}m$ structure from decomposition as a function of pressure, using the variable-cell nudged elastic band method^22^. For BaSiH_8_, for example, we found a *p*_kin_ of ~30 GPa, significantly higher than the dynamical value *p*_dyn_ = 3 GPa.

It was argued in a recent work^23^ that quantum lattice effects treated within the stochastic self-consistent harmonic approximation (SSCHA) drastically increase the dynamical stabilization pressure *p*_dyn_ for LaBH_8_ and it was further suggested that a similar increase in *p*_dyn_ should be expected for other $Fm\bar{3}m$* XY*H_8_ hydrides.

To investigate this, we apply the SSCHA formalism to BaSiH_8_, which, so far, has the lowest *p*_dyn_ among all $Fm\bar{3}m$ *XY*H_8_ hydrides. In SSCHA, a major bottleneck is represented by the need to use large supercells and large numbers of randomly displaced structures if one wants to fully converge the calculation. We overcome this problem by employing machine-learned moment tensor potentials (MTP)^24,25^ that allow us to obtain total energies, forces, and stresses with density-functional-theory (DFT) accuracy but at a fraction of the computational cost^26–28^. To our knowledge, this work represents the first combination of MTPs with the SSCHA method. In addition, we introduce a method to discern the contribution of quantum ionic (QI) effects from those of anharmonic (anh) and phonon-phonon (ph-ph) effects.

We find that *p*_dyn_ increases from 3 GPa to about 20 GPa within the SSCHA and that this rise can almost entirely be attributed to QI effects, with actual anharmonic and ph-ph effects playing only a subordinate role. In fact, the same crystal structure that minimizes the free energy within SSCHA can already be obtained at the harmonic level using DFT by including zero-point energies (ZPE).

Particularly, we demonstrate that even after including QI, anharmonic, and ph-ph effects within the framework of SSCHA, the actual limit of stability is still set by *p*_kin_ (~30 GPa), as stated in our previous work^19^.

## Results

### Ab-initio machine-learned interatomic potentials

In the self-consistent harmonic approximation, the system of fully anharmonic and interacting lattice vibrations is mapped onto an auxiliary harmonic system and the free energy ${{{{{{{\mathcal{F}}}}}}}}$ of the full system is approximated by the minimum of the free energy of the auxiliary harmonic system^29–32^. In the SSCHA, this minimization is performed stochastically via Monte Carlo summation and importance sampling over several consecutive ensembles (populations) of a large number of individuals. Each individual here corresponds to a supercell structure with displaced atomic positions, where the supercell size determines the density of phonon wave vectors in the Brillouin zone^33^. More details are provided in the Method section, in Supplementary Method 2, and in refs. ^34–40^.

In practice, to calculate accurate phonon frequencies within SSCHA, in particular for slow-converging soft modes, one needs to consider population sizes of several ten or hundred thousands individuals. In addition, one also needs to converge the supercell size.

**Fig. 1:**
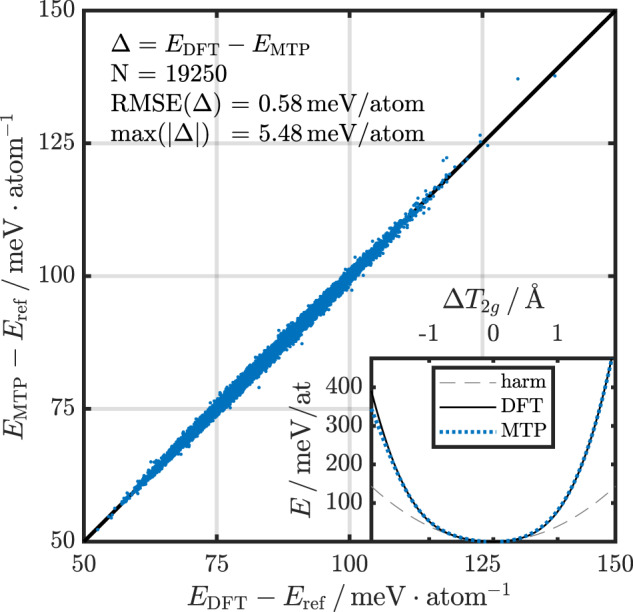
MTP validation. MTP total energy (*E*_MTP_) versus DFT total energy (*E*_DFT_) for all *N* = 19250 individuals of a SSCHA calculation for a lattice constant *a* = 6.242 Å (blue scatter plot). *E*_ref_ is the DFT total energy of the high-symmetry structure with undisplaced H positions. The diagonal black line serves as guide to the eye. The inset shows the full frozen-phonon potential of the lowest *T*_2*g*_ mode at Γ obtained with DFT (solid black line) and MTP (dotted blue line), as well as the harmonic potential (dashed gray line). The root-mean-squared error (RMSE) and the maximum value of the prediction error Δ = *E*_DFT_ − *E*_MTP_ for the presented data set is shown in the upper left corner.

Doing this fully at a DFT level is computationally prohibitive, which is why we made use of MTPs in this work. For every pressure, MTPs were trained on DFT results of 50 structures randomly chosen out of the SSCHA random-displacement individuals in 2 × 2 × 2 supercells. We then validated the trained MTPs for all other individuals by comparing the total energies, forces and stress components. This validation is shown in Fig. 1, demonstrating the exceptional accuracy of the used MTPs (see Supplementary Fig. 2 for other pressures, as well as forces and stresses). As can be appreciated in this figure, the root-mean-squared error (RMSE) is below 1 meV/atom, i.e., at the same level as the error in DFT. The inset also shows that the potential energy surface of the slow-converging, *T*_2*g*_ mode at Γ is reproduced very nicely with the MTPs.

As a final validation, we compare the SSCHA phonon dispersions obtained using only DFT with those employing only MTPs (see Supplementary Fig. 3) and find very good agreement, with only minor differences in the *T*_2*g*_ mode at Γ and the *E*_*g*_ at *X*. To fully converge these modes within 1 meV, we increased the populations sizes within MTP-SSCHA up to 100,000 individuals compared to 10,000 for the DFT-SSCHA calculations.

The use of MTPs does not only substantially speed up the calculations (we found MTPs to be a factor of about 20,000 faster than DFT in the case of 2 × 2 × 2 supercells of BaSiH_8_), but also gives access to larger supercells. In this work, we performed additional SSCHA calculations using MTPs on *n* × *n* × *n* supercells with *n* = 1, 2, 3, 4 at all studied pressures. The convergence of the free energy, the structural parameters, and the phonon dispersions with respect to the supercell size is provided in Supplementary Figs. 4–6. An overview of all performed SSCHA runs is given in Supplementary Tab. 1. Unless stated otherwise, all SSCHA results presented in the following have been obtained with MTPs for 100,000 individuals in 4 × 4 × 4 supercells at 0 K.

### Structural parameters and electronic dispersion

The $Fm\bar{3}m$ phase of BaSiH_8_ has a face-centered cubic unit cell with 10 atoms in the primitive cell, where Ba and Si occupy Wyckoff 4*a*/*b* sites and the H occupy 32*f* sites. The eight H atoms form rhombicuboctahedral cages around the Ba atoms and cubic cages around the Si atoms. The structure has only two free parameters, namely the lattice constant *a* and the Wyckoff coordinate of the 32*f* sites *x*, defining the H-H distance *d*_H-H_ = 2*a* ⋅ *x* (side length of the cubic cage) and the H-Si distance ${d}_{{{{{{{{\rm{H-Si}}}}}}}}}=a\sqrt{3}\cdot x$ (half the space diagonal of the cubic cage).

Relaxing the structure within DFT to target pressures of 10, 20, 25, and 30 GPa, we obtained lattice constants between 6.5 Å and 6.2 Å, and H-Si distances of about 1.6 Å, as shown in Table 1. An extensive list of the structural parameters from ambient pressure up to 100 GPa, as well as the fit to the Birch–Murnaghan equation of state can be found in Supplementary Fig. 1 and Supplementary Note 1.

**Table 1:** Structural parameters.

*a*/Å	*x*	*d*_H-Si_/Å	*p*/GPa	$\tilde{x}$	${\tilde{d}}_{{{{{{{{\rm{H-Si}}}}}}}}}$/Å	$\tilde{p}/{{{{{{{\rm{GPa}}}}}}}}$
6.541	0.1434	1.625	10	0.1459	1.653	12.2
6.323	0.1471	1.611	20	0.1498	1.640	22.6
6.242	0.1483	1.603	25	0.1510	1.633	27.9
6.171	0.1494	1.597	30	0.1521	1.626	33.1

Lattice constant *a*, Wyckoff parameter *x*, H-Si distance *d*_H-Si_, and pressure *p* after the relaxation with respect to the DFT total energy and after the constant-volume relaxation within SSCHA ($\tilde{x},\tilde{d}$, and $\tilde{p}$).

Starting from the atomic positions obtained in DFT and the harmonic dynamical matrices obtained in density-functional perturbation theory (DFPT) calculations at each pressure, we performed constant-volume SSCHA relaxation calculations. The corresponding parameters, indicated by $\tilde{x},\tilde{d}$, and $\tilde{p}$, are reported in Table 1.

**Fig. 2:**
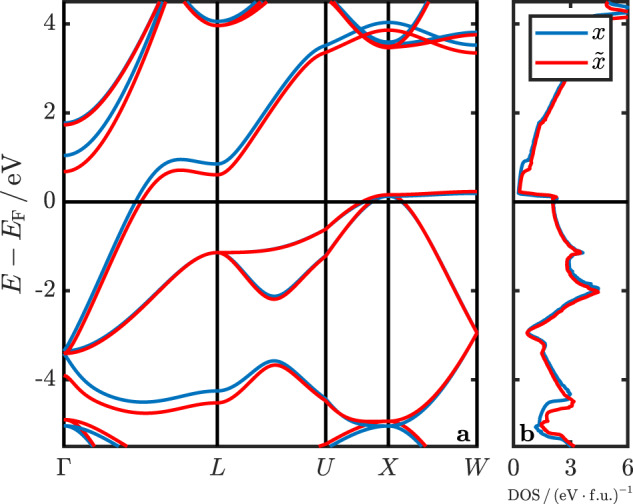
Difference in electronic properties. **a** Electronic bands and **b** density of states for the structure with H positions defined by *x* (DFT minimum, blue line) and $\tilde{x}$ (SSCHA minimum, red line) for *a* = 6.242 Å. The legend in (**b**) also applies to (**a**).

We observe an elongation of *d*_H-H/Si_ of about 30 mÅ (2%) for all pressures and an increase in pressure of about 2 to 3 GPa, i.e., ~20% at 10 GPa and ~10% at 30 GPa. The change in atomic positions introduces only small changes in the electronic structure, as demonstrated in Fig. 2, where we compare the electronic bands and densities of states (DOS) for *x* and $\tilde{x}$. The largest differences are found above and below the Fermi energy, whereas electronic bands and DOS at the Fermi energy, and hence the Fermi surface, remain essentially unchanged.

### Phonon dispersions and lattice instability

**Fig. 3:**
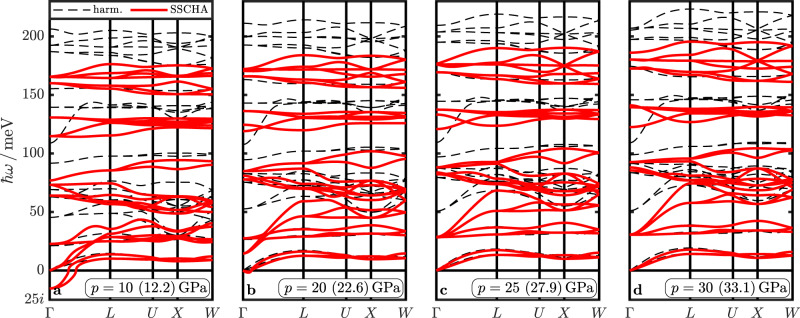
Harmonic and SSCHA phonon dispersions. Phonon dispersions for various pressures along a high-symmetry path of the BZ. The dashed black lines correspond to harmonic calculations and the solid red lines to the SSCHA results. The legend in (**a**) also applies to (**b**–**d**). The pressures indicated in (**a**–**d**) correspond to the DFT (SSCHA) pressures for relaxed atomic positions defined by *x* ($\tilde{x}$). The related values for *x*, $\tilde{x}$, and the lattice constant *a* are reported in Table 1. The small imaginary dip near Γ in (**b**) is due to interpolation, all phonon frequencies at wave vectors commensurate with the supercell are positive.

Moving on, we evaluate and compare the DFPT and SSCHA phonon dispersions at all studied pressures, as shown in Fig. 3. Similar to the results for LaBH_8_ reported by Belli et al.^23^, we find that the high-energy optical modes are strongly renormalized to lower frequencies. In particular, a significant softening occurs for the threefold degenerate *T*_2*g*_ mode at Γ (harmonic values around 50 meV in Fig. 3), which becomes imaginary and indicates a (dynamic) lattice instability for lattice constants *a* > 6.323 Å, corresponding to *p* = 20 GPa and $\tilde{p}$ = 22.6 GPa. Thus, the inclusion of quantum lattice effects within the SSCHA shifts the dynamical stability pressure from the anharmonic frozen-phonon value *p*_dyn_ = 3 GPa to ${\tilde{p}}_{{{{{{{{\rm{dyn}}}}}}}}}$ = 20 GPa (see Supplementary Fig. 7). This ~17 GPa difference is substantial, but considerably smaller than the ~40 GPa shift reported for LaBH_8_^23^.

We want to note in passing that we also calculated the fourth order corrections to the phonon frequencies in SSCHA (cf. Supplementary Method 2) and find, in contrast to the work on LaBH_8_^23^, but in accordance with other works employing SSCHA^35,36,38–40^, only minor differences to the results obtained only up to third order. The maximum phonon energy differences are on the order of about 1 meV for all pressures. The phonon dispersions in the 2 × 2 × 2 supercells with second (auxiliary), third, and fourth order terms for all studied pressures are shown in Supplementary Fig. 8.

### Different effects contributing to frequency shifts

The observed changes in the phonon dispersions when employing SSCHA and the resulting different dynamical stabilization pressures result from a combination of several effects that are not included at the level of standard DFT and DFPT. These are most importantly the vibrational contributions of the ions to the free energy, phonon anharmonicity, and ph-ph interactions. In the following, we will present an attempt to disentangle and determine the importance of each of these effects for BaSiH_8_. Before doing so, however, we want to briefly touch upon terminologies around phonon anharmonicity. Anharmonicity gives rise to ph-ph interactions, but also to the phonon self-interaction of a single mode; effects that are sometimes collectively referred to as anharmonic effects. In a frozen-phonon approach, single-mode potentials are usually obtained without incorporating ph-ph interactions, and there anharmonicity then refers to any deviation from the idealized parabolic potential. For the sake of clarity, we mention both aspects explicitly in the subsequent discussion.

#### QI effects

First, we want to look at the contributions to the total energy originating from the so-called zero-point vibrations, i.e, vibrations of the ions around their equilibrium positions due to the quantum mechanical treatment of the nuclei, absent in the classical, *clamped-nuclei* picture^41^. In the Born–Oppenheimer approximation, the total energy *E*_tot_[**R**] (at *T* = 0 K) for ionic positions **R** is given by the sum of the internal electronic energy *E*_el_[**R**] and the ZPE contributions of the nuclei *E*_ZP_[**R**]. In most solids, *E*_ZP_ is much smaller than *E*_el_ and can be safely neglected. However, due to the small mass of H and the resulting high phonon frequencies in hydrides, the ZPE can become substantial and thus cause a modification of the equilibrium crystal structure.

**Fig. 4:**
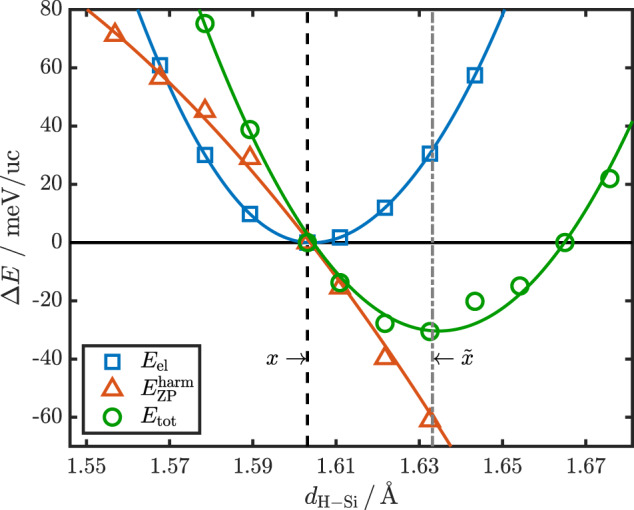
Electronic total energy, harmonic ZPE, and total energy. Electronic total energy *E*_el_ (blue squares), harmonic ZPE ${E}_{{{{{{{{\rm{ZP}}}}}}}}}^{{{{{{{{\rm{harm}}}}}}}}}$ (red triangles), and resulting total energy *E*_tot_ (green circles) as a function of H-Si distance for *a* = 6.242 Å, where the DFT minimum *x* and the SSCHA minimum $\tilde{x}$ are marked explicitly, the latter coinciding with the minimum position of *E*_tot_. The three energy curves are plotted relative to their respective values at *x*, i.e., ${{\Delta }}E=E{| }_{{d}_{{{{{{{{\rm{H-Si}}}}}}}}}}-E{| }_{x}$. The solid lines represent a cubic spline for *E*_el_ and second order polynomial fits for the other energies.

At the harmonic level, the true ZPE can be approximated via ${E}_{{{{{{{{\rm{ZP}}}}}}}}}[{{{{{{{\bf{R}}}}}}}}]\approx {E}_{{{{{{{{\rm{ZP}}}}}}}}}^{{{{{{{{\rm{harm}}}}}}}}}[{{{{{{{\bf{R}}}}}}}}]=\int\nolimits_{0}^{\infty }{{{{{{{\rm{d}}}}}}}}\omega {\rho }_{{{{{{{{\bf{R}}}}}}}}}(\omega )\hslash \omega /2$, where *ρ*_**R**_(*ω*) is the DFPT phonon density of states and *ℏω*/2 the ZPE of a quantum harmonic oscillator. At constant volume, the only free parameter in the $Fm\bar{3}m$ structure is the Wyckoff parameter *x*, defining the H-Si distance, for which we have plotted *E*_tot_, *E*_el_, and ${E}_{{{{{{{{\rm{ZP}}}}}}}}}^{{{{{{{{\rm{harm}}}}}}}}}$ relative to their respective values at the DFT minimum *x* in Fig. 4 for a lattice constant of *a* = 6.242 Å. The results for other lattice constants, i.e., pressures, are provided in Supplementary Fig. 9. We also want to note at this point that for structures not in the DFT minimum, non-vanishing forces occur, at odds with the underlying harmonic approximation. In all the cases considered here, however, the individual atomic force components are still small enough within our computational setup to result in purely real frequencies and sufficiently accurate estimates for the ZPE (see Supplementary Figs. 11 and 12 for further details).

As can be appreciated in this figure, the inclusion of the ZPE, even at the harmonic level, shifts the position of the minimum of the total energy considerably and puts it almost exactly at the minimum position $\tilde{x}$ predicted by the SSCHA. The differences in *d*_H-Si_ between the SSCHA calculations and the ZPE analysis are, in fact, of the order of 1 mÅ, i.e., well within the observed stochastic noise in SSCHA. We want to note that the same is true for LaBH_8_ (see Supplementary Fig. 10). Furthermore, inclusion of ${E}_{{{{{{{{\rm{ZP}}}}}}}}}^{{{{{{{{\rm{harm}}}}}}}}}[{{{{{{{\bf{R}}}}}}}}]$ reduces the total energy at its minimum by ~30 meV/uc compared to its value at the DFT minimum, agreeing very nicely with the result from SSCHA (~27 meV/uc).

This demonstrates the importance of QI effects of the light H ions on the dynamic stability of the hydride materials, and shows that the minimum structure from SSCHA can already be obtained at the level of harmonic ZPE corrections, at least for this class of materials.

**Fig. 5:**
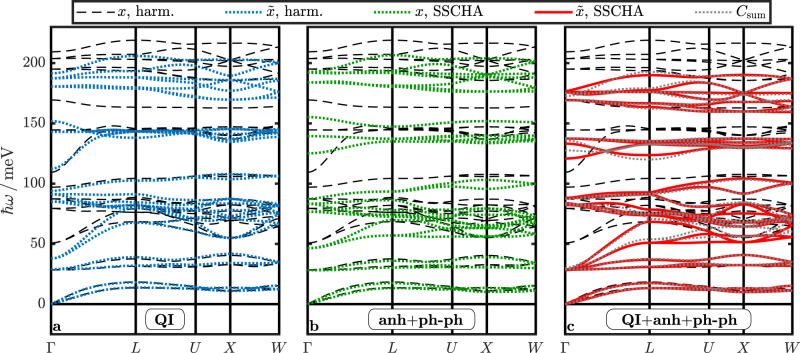
Different effects contributing to shifts in the phonon dispersions. **a** Harmonic dispersion for atomic positions defined by *x* (dashed black) and $\tilde{x}$ (dotted blue). **b** SSCHA-obtained dispersions for fixed atomic positions defined by *x* (dotted green lines) and harmonic reference (dashed black). **c** Dispersions for SSCHA-relaxed atomic positions defined by $\tilde{x}$ (solid red), obtained from *C*_sum_ (dotted gray, see text for more details), and for the harmonic case (dashed black).

Having established that the ZPE has a crucial effect on the structure, we investigate the effect of the changed structure on the phonon dispersions. In Fig. 5a, we present the harmonic dispersions for atomic positions defined by *x* and $\tilde{x}$. We observe large differences for the high-energy optical modes above 150 meV, but also for the low *T*_2*g*_ mode at Γ. The energy shifts for these modes are between 15 and 25 meV.

#### Anharmonicity and ph-ph interaction effects

Having established the QI effects on the structure and the phonon dispersions, we now want to assess the contributions of phonon anharmonicity (anh) and ph-ph interactions. To do that, we perform a SSCHA calculation while keeping the ions fixed at the DFT equilibrium positions, thus qualitatively removing structural effects on the phonon frequencies. In that case, we obtain non-vanishing forces within SSCHA (cf. Supplementary Method 2). As the individual force components on the H atoms are still small (~150 meV/Å in the cell with *a* = 6.242 Å, for example), a fixed-ion calculation seems applicable for a qualitative insight. The phonon dispersions obtained from this calculation are presented in Fig. 5b, where we find that all H-dominated optical modes in the whole BZ experience a sizable frequency renormalization. It is worth noting that fixed-ions SSCHA calculations indicate the onset of dynamical instability just between 5 and 10 GPa (8.4 and 12.2 GPa, respectively, for the SSCHA pressure $\tilde{p}$, see Supplementary Fig. 13), which is reasonably close to the frozen-phonon anharmonic (harmonic) *p*_dyn_ = 3(5) GPa^19^.

#### Combining QI, anh, and ph-ph effects

As we made the attempt of separating the different contributions from QI effects, anharmonicity, and ph-ph interactions, it is tempting to combine the individual contributions to the phonon dispersion as a simple sum and compare it to the full SSCHA calculation. We approach this task in real space via adding the force constants *C*_sum_ = *C*_harm_ + Δ*C*_QI_ + Δ*C*_anh/phph_, where *C*_harm_ is the force constant matrix for harmonic phonons of structure *x*, Δ*C*_QI_ are the force constant contributions due to the structural changes based on including the ZPE (QI effects), and Δ*C*_anh/phph_ are the force constant contributions to the anh and ph-ph interaction effects (see Supplementary Note 3).

The dispersions obtained from *C*_sum_ are shown in Fig. 5c, where we also present as reference the phonon dispersions from a full SSCHA calculation. Again, we find very good agreement between these results, providing further, a posteriori justification and support for the qualitatively introduced separation Ansatz. In Table 2, we summarize the considered effects, methods, and the corresponding physical description of the ions.

**Table 2:** Overview of ionic treatment.

${}_{\downarrow {{{{{{{\rm{structure}}}}}}}}}^{{{{{{{{\rm{phonon}}}}}}}}\to }$	DFPT	SSCHA
*x* $\hat{=}$ min(*E*_el_)	Classical ions	Interact. quantum ions
DFT	in el. GS	in el. GS
	(standard)	(anh+ph-ph)
$\tilde{x}$ $\hat{=}$ min(*E*_tot_)	Quantum ions	Interact. quantum ions
ZPE/SSCHA	in lattice GS	in lattice GS
	(QI)	(QI+anh+ph-ph)

The separate cases are classified according to the structural and phonon treatment. The phonons are obtained either via DFPT or SSCHA. The ground-state (GS) structure is determined by minimizing either the electronic energy within DFT or the total energy including the ZPE (using DFPT or SSCHA).

### Superconductivity

As BaSiH_8_ is potentially a very promising high-*T*_c_ superconductor, we also want to assess the implications of the above mentioned effects on its superconducting (SC) properties. To do that, we solved the anisotropic Migdal–Eliashberg (ME) equations as implemented in EPW^42^ for the four cases in Table 2. Details about the calculation within EPW are provided in the Method section and in Supplementary Method 3, at this point we only want to highlight that for each case we used the corresponding force constants to compute the dynamical matrices, and computed the electron–phonon (*ep*) coupling matrix elements as the self-consistent first-order variation of the potential using the equilibrium positions as defined in Table 2. In Table 3, we summarize the obtained values for quantities characterizing the SC state, i.e., the *ep* coupling strength *λ*, the logarithmic average of the phonon frequencies ${\omega }_{\log }$, and the SC critical temperature *T*_c_.

**Table 3:** SC properties from ME theory.

Effect	(struct., phon.)	ω_log_/meV	λ	*T*_c_/K
Standard	(*x*, harm.)	54	1.25	84
QI	($\tilde{x}$, harm.)	47	1.43	82
anh+ph-ph	(*x*, SSCHA)	54	1.38	90
QI+anh+ph-ph	($\tilde{x}$, SSCHA)	28	2.12	94

Critical temperature *T*_c_, *ep* coupling strength *λ*, and logarithmic phonon frequency average ${\omega }_{\log }$ for the cases discussed in the text.

The corresponding Eliashberg spectral functions *α*^2^*F*(*ω*) and the cumulative coupling strengths *λ*(*ω*) are shown in Supplementary Fig. 14. We want to stress that the provided values for *T*_c_ are obtained by the solution of the full ME equations. The distribution of the SC gap function Δ_**k**_ indicates no change in the distinct two-gap shape calculated for the pure harmonic case^19^. The differences in ${\omega }_{\log }$, *λ*, and *T*_c_ are in the order of 10-15% except for the full SSCHA calculation, where we see a considerable increase in *λ* to almost double the harmonic value, but also a decrease in ${\omega }_{\log }$, compensating the enhancement of *λ*. The resulting *T*_c_ is increased from 84 to 94 K, showing that the full inclusion of all discussed effects results only in ~10-15% change in *T*_c_ for BaSiH_8_.

## Discussion

In this work, we study the effects of quantum lattice dynamics within the SSCHA framework on the structure and the dynamical stability of the $Fm\bar{3}m$ phase of BaSiH_8_. The SSCHA structure relaxation suggests a 2% elongation of the H-H and H-Si bonds for the studied pressure range of 10 to 30 GPa (~30 mÅ).

In the phonon dispersions, we find an overall softening of the high optical modes, as well as a dynamic lattice instability characterized by imaginary SSCHA phonon frequencies in the *T*_2*g*_ mode at Γ below 20 GPa, setting the estimate for the critical dynamical pressure to ${\tilde{p}}_{{{{{{{{\rm{dyn}}}}}}}}}\approx$ 20 GPa. We have further demonstrated the importance of QI effects over anharmonicity and ph-ph interactions, and found that the change in structure, and consequently in pressure, can already be understood by considering harmonic ZPE corrections to the total electronic energy of the system alone (which can be obtained much faster than performing a full SSCHA calculation, cf. Supplementary Note 4). We want to stress at this point that while the total energy ground-state can be obtained already within the harmonic theory, quantum ionic effects and anharmonicity have to be taken into account to accurately determine the corresponding dynamical critical pressure.

We are now left with the question: what is the stability boundary of $Fm\bar{3}m$-BaSiH_8_? In our previous work on BaSiH_8_^19^, we challenged the common practice of assuming the range of metastability of high-pressure hydride phases to coincide with the range of (an)harmonic dynamical stability, which systematically underestimates the stabilization pressures needed to synthesize these materials in reality^43–46^. Dynamical stability is only a prerequisite for thermodynamic metastability, which is characterized by the existence of a distinctive enthalpy barrier that protects a metastable phase from decomposition into other phases (kinetic stability). In our previous work^19^, we calculated the enthalpy transition path to the thermodynamic groundstate at different pressures (corresponding to a decomposition of the $Fm\bar{3}m$ BaSiH_8_ phase into BaSiH_6_ + H_2_ in molecular form), and could estimate the barrier height from the intermediate structures (cf. Supplementary Fig. 16, where we also report the minor influence of ZPE on the determined barrier heights). In combination with the calculated convex hulls for the B-S-H system, we can argue with confidence that the $Fm\bar{3}m$ BaSiH_8_ phase could be synthesized above 100 GPa, and retained down to ~30 GPa, where a distinctive enthalpy barrier still exists. At lower pressures, metastable $Fm\bar{3}m$ BaSiH_8_ will decompose, even though (anharmonic) lattice dynamics calculations predict it to be stable. Hence, kinetic stability poses a stricter bound for synthesizability than dynamical stability.

In conclusion, employing ab-initio machine-learned MTPs, we were able to perform SSCHA calculations for BaSiH_8_ at various pressures for supercells up to 4 × 4 × 4 and more than 100,000 individuals. The inclusion of QI effects, anharmonicity, and ph-ph interactions within the SSCHA-framework increases the pressure of dynamical stability from *p*_dyn_ ≈ 3 GPa to ${\tilde{p}}_{{{{{{{{\rm{dyn}}}}}}}}}\approx$ 20 GPa. We identified the change in structure due to QI effects to be the main driving force here, something that can already be captured to good approximation at the level of harmonic zero-point energy corrections.

Most importantly, the determined ${\tilde{p}}_{{{{{{{{\rm{dyn}}}}}}}}}\approx$ 20 GPa is still below *p*_kin_ ≈ 30 GPa posed by the concept of kinetic stability, thus the latter represents a much stricter bound for the stability and realizability in these materials.

## Methods

### DF(P)T calculations

All DFT and DFPT calculations of electronic and vibrational properties were carried out using the plane-wave pseudopotential code QUANTUM ESPRESSO^47^, scalar-relativistic optimized norm-conserving Vanderbilt pseudopotentials^48^, and the PBE-GGA exchange and correlation functional^49^. The unit cell calculations are done in the face-centered cubic primitive unit cell with 10 atoms, a 12 × 12 × 12 **k**-grid, and a plane-wave cutoff energy of 80 Ry. The 2 × 2 × 2 supercell calculations were done on a 6 × 6 × 6 **k**-grid. Further details are provided in Supplementary Method 1.

### SSCHA calculations

The calculations in the SSCHA are done in the constant-volume relaxation mode, i.e., minimizing the free energy with respect to the average atomic positions ${{{{{{{\mathcal{R}}}}}}}}$ and the force constants Φ, as implemented in the SSCHA python package^40^. We use the DFT equilibrium atomic positions and the DFPT dynamical matrices on a 2 × 2 × 2 **q**-grid as initial guesses for ${{{{{{{\mathcal{R}}}}}}}}$ and Φ, respectively. The starting point for the larger supercells is obtained by interpolating the previously converged auxiliary dynamical matrices.

The total energies, forces, and stress tensors for the individuals are obtained from DFT calculations or from machine-learned interatomic potentials in the framework of MTPs, see below. At the end of a minimization run, a new population with higher number of individuals *N* is generated from the minimized trial density matrix until convergence. We set two stopping criteria for the minimization loops: a Kong–Liu ratio for the effective sample size of 0.2, and ratio of <10^−7^ between the free energy gradient with respect to the auxiliary dynamical matrix and its stochastic error. In calculations based on DFT we increased *N* up to 10^4^ individuals, for the MTP cases up to 10^5^.

The anharmonic phonon dispersions are obtained from the positional free-energy Hessians without the fourth-order term, if not specified otherwise explicitly. The final atomic positions are obtained from the converged average atomic positions ${{{{{{{\mathcal{R}}}}}}}}$ and the pressure as derivative of the converged free energy with respect to a strain tensor. The free energy difference between the last two populations in the 2 × 2 × 2 cells is well below 1 meV/uc, and well below 0.1 meV/uc for higher cells. The total forces in the last population are well below 10^−6^ meV/Å. The physical phonon frequency differences between the last two populations are below 5 meV for the DFT cases and well below 1 meV for the MTP case (*T*_2*g*_ and *A*_2*u*_ converge slower, see Supplementary Fig. 6). All calculations were carried out at zero temperature. We note in passing that with these settings, we could reproduce all LaBH_8_ results from Belli et al.’s work^23^.

Further details are provided in Supplementary Method 2 and details on convergence of the free energy, its gradients, and the auxiliary frequencies in a SSCHA calculation are shown in Supplementary Fig. 15.

### Moment tensor potentials

The MTPs were trained and evaluated using the MLIP package^24,50^. We choose a functional form of level 26, eight radial basis functions, *R*_cut_ = 5.0 Å and ${R}_{\min }$ = 1.2 Å, and trained on 50 structures in a 2 × 2 × 2 supercell randomly chosen out of all individuals of the DFT SSCHA calculations. We trained separate MTPs for each set of pressure to ensure the highest possible accuracy of our calculations. We validated the potentials on all individuals in the DFT SSCHA calculations and find a RMSE on the total energy of 0.5–0.6 meV/atom, 45–50 meV/Å for the force components, and 0.3–0.4 GPa for the diagonal stress tensor components. We further validated the MTPs on 30 randomly chosen individuals in a 3 × 3 × 3 supercell and achieve similar RMSEs. The validations and RMSEs for each pressure are shown in Supplementary Fig. 2 and Supplementary Note 2.

### ZPE and total energy

The internal electronic energy *E*_el_[**R**] is obtained from DFT calculations at fixed volume by varying the H-Si distance via the Wyckoff parameter *x* of the H positions. The phonon density of states *ρ*_**R**_(*ω*) is obtained using DFPT on a 2 × 2 × 2 **q**-grid, interpolated on a 16 × 16 × 16 **q**-grid. Smooth ZPE and total energy curves are obtained by second-order polynomial fits in the H-Si distance. Due to the shift out of the DFT equilibrium structure, forces on the individual H atoms arise at the DFT level. Around the total energy minimum, the force components are in the order of 150 meV/Å, i.e., small enough to warrant the use of linear-response theory to gain qualitative and systematic insights. DFT diagonal stress tensor components (pressures) are decreased by about 2 GPa in the shifted structures around the total energy minimum. The small non-zero forces do not lead to imaginary phonon frequencies in any DFPT calculation for BaSiH_8_ with varied H-Si distance.

Note on similarities to the quasi-harmonic approximation (QHA): Within the QHA, the temperature-dependent internal vibrational energy and the vibrational entropy are commonly added to a system to study temperature effects. The standard QHA only varies external coordinates, such as the volume, but keeps atomic positions in the DFT minimum. In our approach to QI effects, we vary explicitly the internal coordinates at fixed volume, instead. Incidentally, the full optimization of the quasi-harmonic free energy with respect to all degrees of freedom within QHA has just been reported^51^.

### ME theory

The Wannier interpolation of the *ep* matrix elements onto dense **k**- and **q**-grids, and the subsequent self-consistent solution of the fully anisotropic ME equations were done in EPW^42,52^, for all the cases in Table 3. We used coarse 6 × 6 × 6 and fine 30 × 30 × 30 **k**- and **q**-grids, a Matsubara frequency cutoff of 1 eV, and a Morel–Anderson pseudopotential μ* = 0.10. The *ep* coupling strength $\lambda =2\int\nolimits_{0}^{\infty }{{{{{{{\rm{d}}}}}}}}\omega \frac{{\alpha }^{2}F(\omega )}{\omega }$ and the logarithmic average phonon frequency ${\omega }_{\log }=\exp \left(\frac{2}{\lambda }\int\nolimits_{0}^{\infty }{{{{{{{\rm{d}}}}}}}}\omega \frac{{\alpha }^{2}F(\omega )\ln \omega }{\omega }\right)$ are obtained from the Eliashberg spectral function *α*^2^*F*(*ω*). Further details are provided in Supplementary Method 3.

## Supplementary information

Supplementary Information

## Data Availability

The authors confirm that the data supporting the findings of this study are available within the article and its Supplementary Materials. Further information is available upon request.
