# Supplementary material for: Quantum lattice dynamics and their importance in ternary superhydride clathrates
Source: Commun Phys. 2023 Oct 16;6(1):298. doi: 10.1038/s42005-023-01413-8 (PMC11549051; doi:10.1038/s42005-023-01413-8)
Supplement: Supplementary file 1 — Supplementary Information [file 42005_2023_1413_MOESM1_ESM.pdf]

Supplemental Material
for
‘Quantum lattice dynamics and their importance in ternary superhydride clathrates’

Roman Lucrezi,[1] Eva Kogler,[1] Simone Di Cataldo,[1,2]
Markus Aichhorn,[1] Lilia Boeri,[2,3] and Christoph Heil[1,†]

[1]*Institute of Theoretical and Computational Physics, Graz University of Technology, NAWI Graz, 8010 Graz, Austria*
[2]*Dipartimento di Fisica, Sapienza Università di Roma, 00185 Rome, Italy*
[3]*Enrico Fermi Research Center, Via Panisperna 89 A, 00184, Rome, Italy*

(Dated: September 22, 2023)

I. SUPPLEMENTARY METHODS

Supplementary Method 1. DFT and DFPT calculations

All density-functional theory (DFT) and density-functional perturbation theory (DFPT) calculations of electronic and vibrational properties were carried out using the plane-wave pseudopotential code Quantum Espresso (QE) [1], scalar-relativistic optimized norm-conserving Vanderbilt pseudopotentials (ONCV) [2], and the PBE-GGA exchange and correlation functional [3]. The unit cell calculations are done in the *fcc* primitive unit cell with 10 atoms, a 12×12×12 **k**-grid, and a plane-wave cutoff energy of 80 Ry. The 2×2×2 supercell calculation were done on a 6×6×6 **k**-grid.

We set the electronic convergence threshold to 10^{-10} Ry, and the phonon self-convergency threshold to 10^{-16}. The relaxation thresholds for the BFGS steps were 10^{-7} Ry in total energy and 10^{-6} Ry/a_0 for all force components. We verified the electronic convergence with respect to a reference calculation with a 40×40×40 Monkhorst-Pack **k**-grid and a kinetic energy cutoff of 300 Ry. With a 12×12×12 **k**-grid, a cutoff of 80 Ry, and a smearing of 0.01 Ry, the changes in total energy are well below 1 meV/atom. For the sake of simplicity, we kept the 12×12×12 **k**-grid for all pressures, resulting in **k** spacings of $|\Delta\mathbf{k}| = 0.021$ to $0.026{\cdot}2\pi$ Å^{-1}. We verified the harmonic phonon convergence on the basis of the first optical mode (T_{1u}) at Γ with respect to a reference calculation with a 30×30×30 Monkhorst-Pack **k**-grid, a kinetic energy cutoff of 300 Ry, and a phonon self-convergency threshold of 10^{-18}. With the electronic parameters above and a phonon threshold of 10^{-16}, the changes in the T_{1u} phonon frequencies are well below 0.1 meV.

Supplementary Method 2. SSCHA calculations

In the stochastic self-consistent harmonic approximation (SSCHA), the system of fully anharmonic and interacting lattice vibrations is mapped onto auxiliary harmonic system in a real space supercell [4–8], with trial density matrix $\tilde{\rho}_{\mathcal{R},\Phi}$ with average atomic positions $\mathcal{R}$, and the dynamical matrix Φ of the chosen supercell [8]. The free-energy functional is then written as

$$\mathcal{F}[\tilde{\rho}_{\mathcal{R},\Phi}] = E[\tilde{\rho}_{\mathcal{R},\Phi}] - TS[\tilde{\rho}_{\mathcal{R},\Phi}], \tag{1}$$

where T is the temperature, S is the entropy of the auxiliary harmonic system, and

$$E[\tilde{\rho}_{\mathcal{R},\Phi}] = \langle K + E_{\mathrm{el}}[\mathbf{R}]\rangle_{\tilde{\rho}_{\mathcal{R},\Phi}} \tag{2}$$

is the sum of the internal electronic and nuclear energies (kinetic energy K and Born-Oppenheimer potential energy surface $E_{\mathrm{el}}[\mathbf{R}]$) evaluated as the expectation value of the state described by the trial density matrix. The free energy $\mathcal{F}$ is minimized iteratively with respect to $\mathcal{R}$ and Φ, and the expectation values of observables are evaluated as Monte-Carlo averages

$$\langle O(\mathbf{R})\rangle_{\tilde{\rho}_{\mathcal{R},\Phi}} = \left(\sum_{j=1}^{N} \rho_j\right)^{-1} \sum_{i=1}^{N} \rho_i O(\mathbf{R}_i), \tag{3}$$

† Corresponding author; christoph.heil@tugraz.at

where $O(\mathbf{R}_i)$ is an observable for a specific ionic configuration $\mathbf{R}_i$ (*individual*), and ρ_i a weighting factor obtained via importance sampling during the minimization. The weights are calculated as $\rho_i = \tilde{\rho}_{\mathcal{R},\Phi}(\mathbf{R}_i)/\tilde{\rho}_{\mathcal{R}',\Phi'}(\mathbf{R}_i)$, and updated during the minimization as $\mathcal{R}$ and Φ change. A suitable ensemble (*population*) of N individuals for the Monte-Carlo summation is drawn from the probability distribution $\tilde{\rho}_{\mathcal{R}',\Phi'}(\mathbf{R}) = \langle \mathbf{R}|\tilde{\rho}_{\mathcal{R}',\Phi'}|\mathbf{R}\rangle$, where $\mathcal{R}'$ and Φ' are fixed for each population. The minimization on one population is stopped if it is no longer sufficiently described by the new density matrix (quantified by the Kong-Liu ratio for the effective sample size $N_{\text{eff}} = (\sum_i \rho_i^2)/(\sum_i \rho_i)^2$) and a new population is drawn, or convergence is achieved. For the first population, $\mathcal{R}'$ and Φ' represent the initial guesses, for all others the relaxed parameters of the previous population.

In DFPT, the phonon frequencies are obtained as the square root of the eigenvalues of the Fourier transform of the real-space dynamical matrix

$$D_{ab}^{\text{DFPT}} = \frac{1}{\sqrt{M_a M_b}} \left.\frac{\partial^2 E_{\text{el}}[\mathbf{R}]}{\partial \mathbf{R}_a \partial \mathbf{R}_b}\right|_{\mathbf{R}_i=\mathbf{R}_0}, \tag{4}$$

where $\mathbf{R}_0$ is the DFT minimum structure, and M_i the atomic masses. Analogous to that, the physical phonon frequencies within the SSCHA (that are in general not the auxiliary phonon frequencies) are obtained as the square root of the eigenvalues of the Fourier transform of the positional free-energy Hessian

$$D_{ab}^{\mathcal{F}} = \frac{1}{\sqrt{M_a M_b}} \left.\frac{\partial^2 \mathcal{F}}{\partial \mathcal{R}_a \partial \mathcal{R}_b}\right|_{\mathcal{R}=\mathcal{R}_{\text{eq}}} \tag{5}$$

evaluated at the positions $\mathcal{R}_{\text{eq}}$ obtained at the end of the minimization. The second derivative of the free energy can be further written as

$$\frac{\partial^2 \mathcal{F}}{\partial \mathcal{R} \partial \mathcal{R}} = \mathbf{\Phi}^{(2)} + \mathbf{\Phi}^{(3)}\mathbf{\Lambda}\left[\mathbf{1} - \mathbf{\Phi}^{(4)}\mathbf{\Lambda}\right]^{-1}\mathbf{\Phi}^{(3)}, \tag{6}$$

where

$$\Phi^{(n)}_{a_i \dots a_n} = \left\langle \frac{\partial^n E_{\text{el}}[\mathbf{R}]}{\partial \mathcal{R}_{a_1} \dots \partial \mathcal{R}_{a_n}} \right\rangle_{\tilde{\rho}_{\Phi,\mathcal{R}}} \tag{7}$$

and $\mathbf{\Lambda}$ is a fourth-order tensor containing the eigenvalues and eigenvectors of the auxiliary system [9]. A comparison of the different contributions $\Phi^{(n)}$ is shown in Supplementary Fig. 8 for a 2×2×2 supercell. Calculating fourth-order corrections on larger supercells is computationally unfeasible with our currently available resources. This calculation in an $n \times n \times n$ supercell requires 4D arrays with $(3 \cdot n^3 \cdot <\text{number of atoms in uc}>)^4$ elements. For 10 atoms in the unit cell and $n = 3$, this yields 810^4 double-precision entries, for which 3.13 TB of RAM are needed on a single shared-memory node.

The forces $\mathbf{F}_a$ and the stress tensor $P_{\alpha\beta}$ are obtained as

$$\mathbf{F}_a = -\frac{\partial \mathcal{F}}{\partial \mathcal{R}_a} \qquad \text{and} \qquad P_{\alpha\beta} = -\frac{1}{V_{\text{sc}}} \left.\frac{\partial \mathcal{F}}{\partial \varepsilon_{\alpha\beta}}\right|_{\varepsilon=0}, \tag{8}$$

where V_{sc} is the supercell volume, $\mathcal{F}$ the free energy functional, and $\varepsilon_{\alpha\beta}$ the strain tensor [10]. The pressure is then obtained as $\tilde{p} = \sum_\alpha P_{\alpha\alpha}/3$.

Performed SSCHA calculations: In Supplementary Tab. 1, we give a comprehensive list of the methods, supercell sizes and number of individuals used in each calculation.

Supplementary Table 1: **Details for performed SSCHA calculations**: DFT pressure p, structure relaxation enabled/disabled (i.e. update $\mathcal{R}$ true [T] / false [F]), supercell size $n \times n \times n$ with 10, 80, 270, and 640 atoms for $n = 1, 2, 3, 4$, respectively, and number of individuals in consecutive populations.

p / GPa	$\mathcal{R}$	method	n	populations and number of individuals	total number
5	F	DFT	2	100, 100, 100, 200, 500, 1000, 2000, 5000	9000
10	F	DFT	2	50, 100, 200, 500, 1000, 2000	3850
	T	DFT	2	50, 100, 100, 100, 200, 200, 500, 500, 1000, 2000	4750
	T	MTP	1	50, 100, 200, 500, 1000, 2000, 5000, 10 000, 20 000, 50 000, 100 000	188 850
	T	MTP	2	250, 250, 250, 500, 1000, 2000, 5000, 10 000, 20 000, 50000	89 250
	T	MTP	3	1000, 1000, 2000, 5000, 10 000, 20 000, 50 000, 100 000	189 000
	T	MTP	4	2000, 5000, 10 000, 20 000, 50 000, 100 000	187 000
20	T	DFT	2	250, 250, 250, 500, 1000, 2000, 5000, 10000	19 250
	T	MTP	1	50, 100, 200, 500, 1000, 2000, 5000, 10 000, 20 000, 50 000, 100 000	188 850
	T	MTP	2	250, 250, 250, 250, 500, 1000, 2000, 5000, 10 000, 20 000, 50000	89 500
	T	MTP	3	1000, 2000, 5000, 10 000, 20 000, 50 000, 100 000	188 000
	T	MTP	4	2000, 5000, 10 000, 20 000, 50 000, 100 000	187 000
25	T	DFT	2	250, 250, 250, 500, 1000, 2000, 5000, 10000	19 250
	T	MTP	1	50, 50, 100, 200, 500, 1000, 2000, 5000, 10 000, 20 000, 50 000, 100 000	188 900
	T	MTP	2	250, 250, 250, 500, 1000, 2000, 5000, 10 000, 20 000, 50000	89 250
	T	MTP	3	1000, 2000, 5000, 10 000, 20 000, 50 000, 100 000	188 000
	T	MTP	4	2000, 5000, 10 000, 20 000, 50 000, 100 000	187 000
30	T	DFT	2	250, 250, 250, 500, 1000, 2000, 5000, 10000	19 250
	T	MTP	1	50, 50, 100, 200, 500, 1000, 2000, 5000, 10000	18 900
	T	MTP	2	250, 250, 250, 500, 1000, 2000, 5000, 10 000, 20 000, 50 000	89 250
	T	MTP	3	1000, 2000, 5000, 10 000, 20 000, 50 000, 100 000	188 000
	T	MTP	4	2000, 5000, 10 000, 20 000, 50 000, 100 000	187 000

Workflow for the DFT and MTP based SSCHA calculations: As initial values for $\mathcal{R}$ and Φ we provided the harmonic dynamical matrices on a 2×2×2 **q**-grid for each (DFT) pressure p. The SSCHA calculations consist of the following steps:

1. Create N individuals in a 2×2×2 supercell. We chose the starting N as roughly the number of phonon modes in the supercell.
2. Calculate total energies, stresses and forces for all individuals.
3. Run minimization (and relaxation) until: (i) the Kong-Liu ratio is below 0.2, or (ii) it converges with a ratio of $< 10^{-7}$ between the free energy gradient with respect to the auxiliary dynamical matrix and its stochastic error.
4. Use the obtained auxiliary dynamical matrices as new starting point
5. Loop step 1-4 with a new population and the same N for case (i), or increase N for case (ii)

6. Post-processing step: calculation of the positional free energy Hessian and convergence check on the physical phonon frequencies.

Step 2 is done with DFT (QE) or MTPs (see Supplementary Tab. 1). The potentials for the MTP cases were trained on 50 individuals in the 2×2×2 supercell randomly chosen from all populations in the DFT case. We followed the same workflow for the higher supercell calculations, but instead of using the harmonic dynamical matrices on the higher grid as an initial guess, we interpolated the final SSCHA auxiliary matrices of smaller supercells to the desired larger supercell. We used the CellConstructor [8] routine `cellconstructor.Phonons.Interpolate` without support dynamical matrices in order to start from the SSCHA relaxed structure and to speed up the convergence.

Supplementary Method 3. Anisotropic Migdal-Eliashberg (ME) equations

The Wannier interpolation of the electron-phonon (*ep*) matrix elements $g_{mn}^{\nu}(\mathbf{k},\mathbf{q})$ onto dense **k**- and **q**-grids, and the subsequent self-consistent solution of the fully anisotropic ME equations were done in Epw [11, 12] for the cases shown in Tab. III in the main text. The *ep* matrix elements can be calculated by evaluating the expectation value $\langle\psi_{m\mathbf{k}+\mathbf{q}}|\left(\partial_{\mathbf{q}\nu}v^{\mathrm{KS}}\right)\cdot\mathbf{e}_{\mathbf{q}\nu}|\psi_{n\mathbf{k}}\rangle$, where $\psi_{n\mathbf{k}}$ is the electronic wavefunction for band n and wavevector $\mathbf{k}$, $\partial_{\mathbf{q}\nu}v^{\mathrm{KS}}$ is the self-consistent first-order variation of the Kohn-Sham potential, and $\mathbf{e}_{\mathbf{q}\nu}$ is the polarization vector for wave vector $\mathbf{q}$, mode index ν, and frequency $\omega_{\mathbf{q}\nu}$. The fully anisotropic Eliashberg function is then given as

$$\alpha^2F(n\mathbf{k},m\mathbf{k}',\omega)=N_{\mathrm{F}}\sum_{\nu}|g_{mn}^{\nu}(\mathbf{k},\mathbf{k}')|^2\,\delta(\omega-\omega_{\mathbf{q}\nu}),\tag{9}$$

where $\mathbf{k}'=\mathbf{k}+\mathbf{q}$ and N_{F} is the DOS at the Fermi energy. The total *ep* coupling strength $\lambda=\lambda(\omega=\infty)$ and the logarithmic average phonon frequency ω_{log} are obtained from the Fermi-surface restricted Eliashberg function

$$\alpha^2F(\omega)=\frac{1}{N_{\mathrm{F}}^2}\sum_{nm}\sum_{\mathbf{k}\mathbf{k}'}\alpha^2F(n\mathbf{k},m\mathbf{k}',\omega)\delta(\varepsilon_{n\mathbf{k}})\delta(\varepsilon_{m\mathbf{k}'}),\tag{10}$$

where $\varepsilon_{n\mathbf{k}}$ is the electronic eigenvalue for $\psi_{n\mathbf{k}}$ with respect to the the Fermi energy E_{F}, as

$$\lambda(\omega)=2\int_0^{\omega}\mathrm{d}\omega'\frac{\alpha^2F(\omega')}{\omega'},\qquad\text{and}\qquad\omega_{\mathrm{log}}=\exp\left(\frac{2}{\lambda}\int_0^{\infty}\mathrm{d}\omega\frac{\alpha^2F(\omega)\ln\omega}{\omega}\right).\tag{11}$$

For the Wannier interpolation of the electronic band structure, we set a frozen window of approximately [-15 eV, +5 eV] around E_{F}, using initial projections onto H s, Si $s,p,d_{z^2},d_{x^2-y^2}$, and Ba p,d orbitals, giving a total of 22 Wannier functions for $BaSiH_8$. For the calculation of λ, ω_{log}, and α^2F, as well as for solving the ME equations, we used 6×6×6 coarse **k**− and **q**-grids, interpolated onto 30×30×30 fine grids. The ME equations are solved independently for a discrete set of temperatures and approaching the critical temperature T_{c}, we used steps of 2 K.

We calculated the harmonic dynamical matrices and the self-consistent first-order variation of the potential on a 6×6×6 **q**-grid, once for the structure with atomic positions defined

by x and once for $\tilde{x}$, and used the files for cases **standard** and **QI** shown in Tab. III in the main text. For case **anh+ph-ph** (SSCHA calculation with atomic positions fixed in x), we replaced the DFPT harmonic matrices with the obtained positional free energy Hessian matrices (interpolated from the 4×4×4 supercell to the 6×6×6 **q**-grid) and retained the potential variation from the calculation in x. For case **QI+anh+ph-ph** (SSCHA calculation with relaxation and final atomic positions in $\tilde{x}$), we replaced the DFPT harmonic matrices with the obtained positional free energy Hessian matrices (interpolated from the 4×4×4 supercell to the 6×6×6 **q**-grid) and retained the potential variation from the calculation in $\tilde{x}$. We note in passing, that a more rigorous treatment would require the replacement of the polarization vectors $\mathbf{e}_{\mathbf{q}\nu}$, as well as either the inclusion of the force term arising in the DF(P)T description of the structure defined by $\tilde{x}$, or an $\mathcal{F}$-based description of the *ep* matrix elements within the SSCHA, which are not implemented yet.

Coulomb effects are included in the ME equations via the Morel-Anderson pseudopotential $\mu^* = 0.10$, as used and described in Ref. [13].

II. SUPPLEMENTARY NOTES

Supplementary Note 1. Structural parameters

The unit-cell calculations are done in the $Fm\bar{3}m$ phase (space group 225) in the *fcc* primitive unit cell with lattice parameter a. Si occupies the Wyckoff position $4a$ (0, 0, 0), Ba the $4b$ (1/2, 1/2, 1/2), and the H atoms the $32f$ (x, x, x). The lattice parameter a and the Wyckoff parameter x are shown in Supplementary Figure 1 over the pressure range from 0 GPa to 100 GPa.

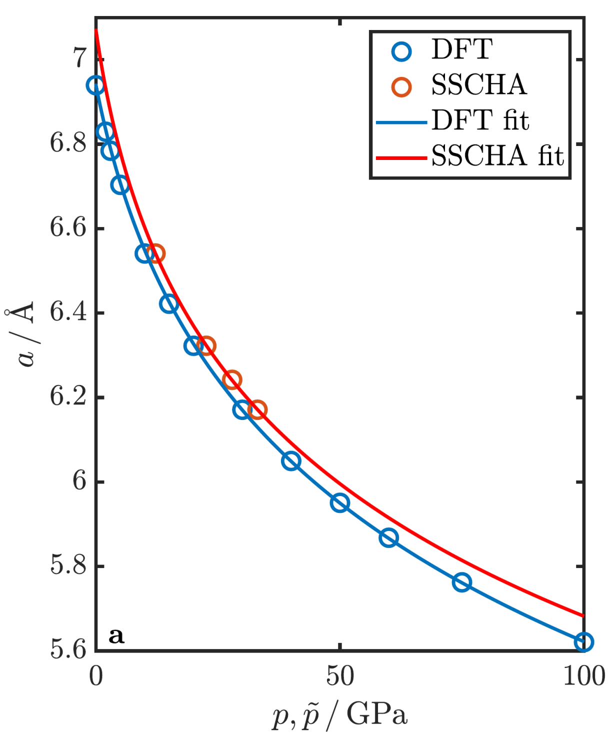

Supplementary Figure 1: **Structural parameters for $BaSiH_8$ within DFT and SSCHA: a** Lattice parameter (circles) and equation-of-state fit (solid line) over pressure calculated within DFT (blue) and SSCHA (red). **b** Hydrogen Wyckoff parameter (x).

We fitted the Birch–Murnaghan equation of state

$$p(V) = \frac{3B_0}{2}\left[\left(\frac{V_0}{V}\right)^{\frac{7}{3}} - \left(\frac{V_0}{V}\right)^{\frac{5}{3}}\right]\left\{1 + \frac{3}{4}\left(B_0' - 4\right)\left[\left(\frac{V_0}{V}\right)^{\frac{2}{3}} - 1\right]\right\} \qquad (12)$$

for the *fcc* unit-cell volumes $V(p)$ and the lattice parameters $a(p)$ (related via $V = a^3/4$) shown in Supplementary Fig. 1, and obtained the fit parameters

$$B_{0;\mathrm{DFT}} = 39.4\,\mathrm{GPa}, \qquad B'_{0;\mathrm{DFT}} = 4.3, \qquad (V_{0;\mathrm{DFT}} = 83.6\,\mathrm{\AA}^3 \text{ fixed from DFT})$$

and

$$B_{0;\mathrm{SSCHA}} = 29.2\,\mathrm{GPa}, \qquad B'_{0;\mathrm{SSCHA}} = 5.0, \qquad V_{0;\mathrm{SSCHA}} = 88.3\,\mathrm{\AA}^3,$$

where B_0 is the bulk modulus, and B_0' its the derivative with respect to pressure.

Supplementary Note 2. MTP validation

In Supplementary Fig. 2, we show the MTP validation and RMSEs for the 2×2×2 supercell and all individuals from the DFT calculations listed in Supplementary Table 1 at all studied pressures. The overall trend in the predicted forces captures the DFT data very well, and outliers cluster around structures with usually very large force components.

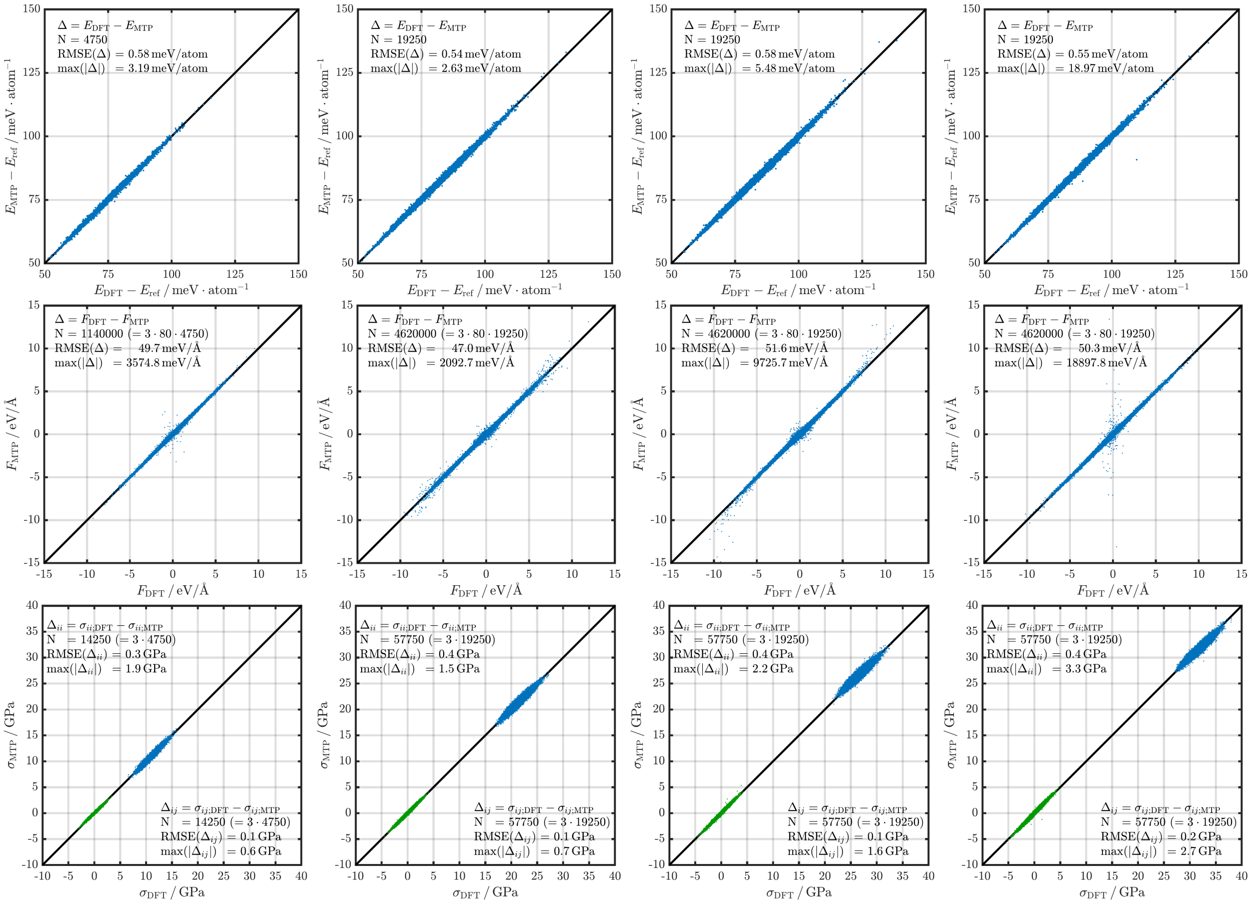

(a) 10GPa (b) 20GPa (c) 25GPa (d) 30GPa

Supplementary Figure 2: **MTP validation:** energy (E), force components (F), and stress tensor components σ calculated with MTPs for all individuals generated in the DFT SSCHA calculations plotted versus the corresponding DFT values. The energies are plotted with respect to the DFT total energy of the undisplaced structure E_{ref} at the indicated pressure. The diagonal elements of the stress tensor σ_{ii} are plotted in blue, the off-diagonal elements $\sigma_{i\neq j}$ in green. The solid black line indicates the exact correlation between MTP and DFT values and serves as a guide to the eye.

In Supplementary Fig. 3, we compare the SSCHA phonon dispersion obtained from DFT and MTPs in 2×2×2 supercells for all studied pressures.

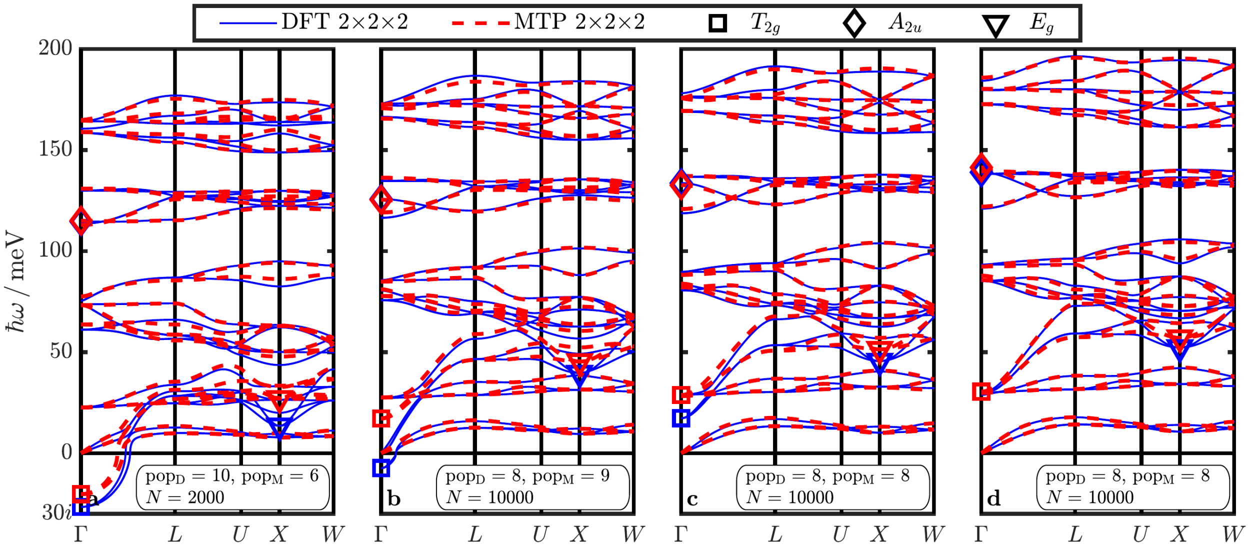

Supplementary Figure 3: **MTP validation in SSCHA:** SSCHA phonon dispersions obtained in the 2×2×2 supercell using DFT (blue solid lines) and MTPs (red dashed lines) in populations with the same number of individuals at **a** $p = 10\,\mathrm{GPa}$, **b** 20 GPa, **c** 25 GPa, and **d** 30 GPa. $\mathrm{pop_D}$ ($\mathrm{pop_M}$) is the population number for the DFT (MTP) case that has the indicated number of individuals N. The squares, diamonds, and triangles mark the slowly-converging modes T_{2g} and A_{2u} at Γ, and E_g at X. See Supplementary Fig. 6 for the convergence with respect to populations.

Supplementary Note 3. Summing the force constants of different contributions

The dotted phonon dispersions in Fig. 5**c** in the main text are obtained from the force constant matrix $C_{\mathrm{sum}} = C_{\mathrm{harm}} + \Delta C_{\mathrm{QI}} + \Delta C_{\mathrm{anh/phph}}$, where C_{harm} is the force constant matrix for harmonic phonons of structure x in the 4×4×4 supercell, and ΔC_{QI} and $\Delta C_{\mathrm{anh/phph}}$ are obtained as the difference between C_{QI}, $C_{\mathrm{anh/phph}}$ and C_{harm}, i.e.

$$\Delta C_{\mathrm{QI}} = C_{\mathrm{QI}} - C_{\mathrm{harm}} \tag{13}$$

$$\Delta C_{\mathrm{anh/phph}} = C_{\mathrm{anh/phph}} - C_{\mathrm{harm}}. \tag{14}$$

C_{QI} and $C_{\mathrm{anh/phph}}$ are the force constant matrices in the 4×4×4 supercell used to create the dotted dispersions in Fig. 5**a** and **b** in the main text.

Supplementary Note 4. Computational costs of the harmonic ZPE analysis

The harmonic analysis is about 50 to 100 times faster than the SSCHA evaluation for $BaSiH_8$. As shown in Supplementary Fig. 6 **a,f,k,b**, convergence for the atomic positions is achieved with populations 4 and 5, corresponding to a total of ∼1500-2000 DFT calculations in a 2×2×2 supercell with 80 atoms (cf. Supplementary Table 1), each of which takes about 10 CPUh, adding up to 15 000-20 000 CPUh. In order to obtain a total energy curve from harmonic theory, we performed 10-20 DFPT calculations in the unit cell (10 atoms) employing 2×2×2 **q**-grids, each of which takes about 20 CPUh, in total about 200-400 CPUh.

III. SUPPLEMENTARY FIGURES

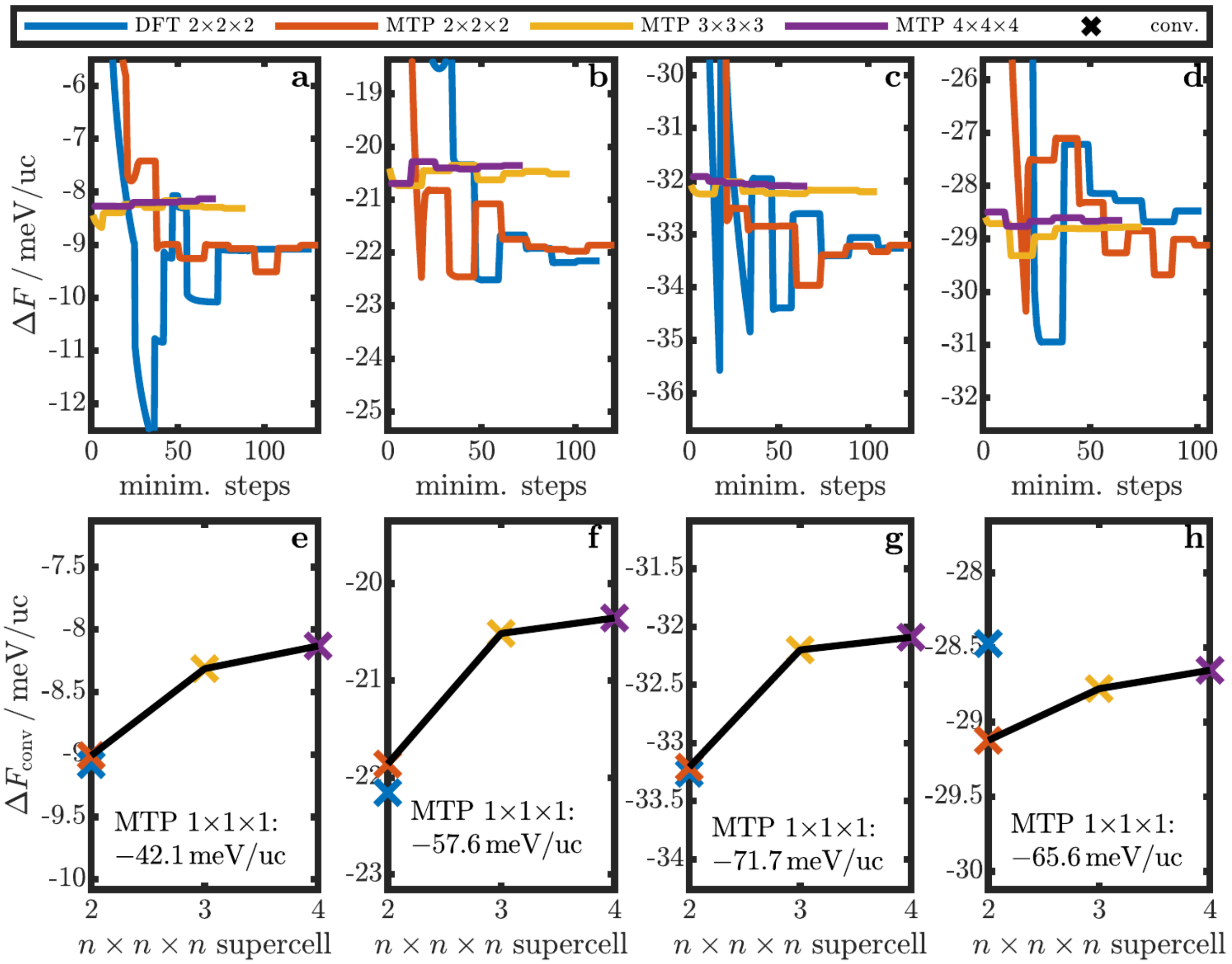

Supplementary Figure 4: **Convergence of the free energy with respect to supercell size: a-d** as a function of minimization steps at $p = 10$, 20, 25 and 30 GPa, **e-h** the converged (last) value of the free energy as a function of the supercell size. The value obtained in the 1×1×1 supercell (unit cell) is shown as text. The free energy is plotted as difference to the DF(P)T harmonic value $\Delta F = \mathcal{F} - (E_{\text{el}}(x) + E_{\text{ZP}}^{\text{harm}}(x))$, where $\mathcal{F}$ if the SSCHA free energy, x defines the DFT relaxed atomic positions.

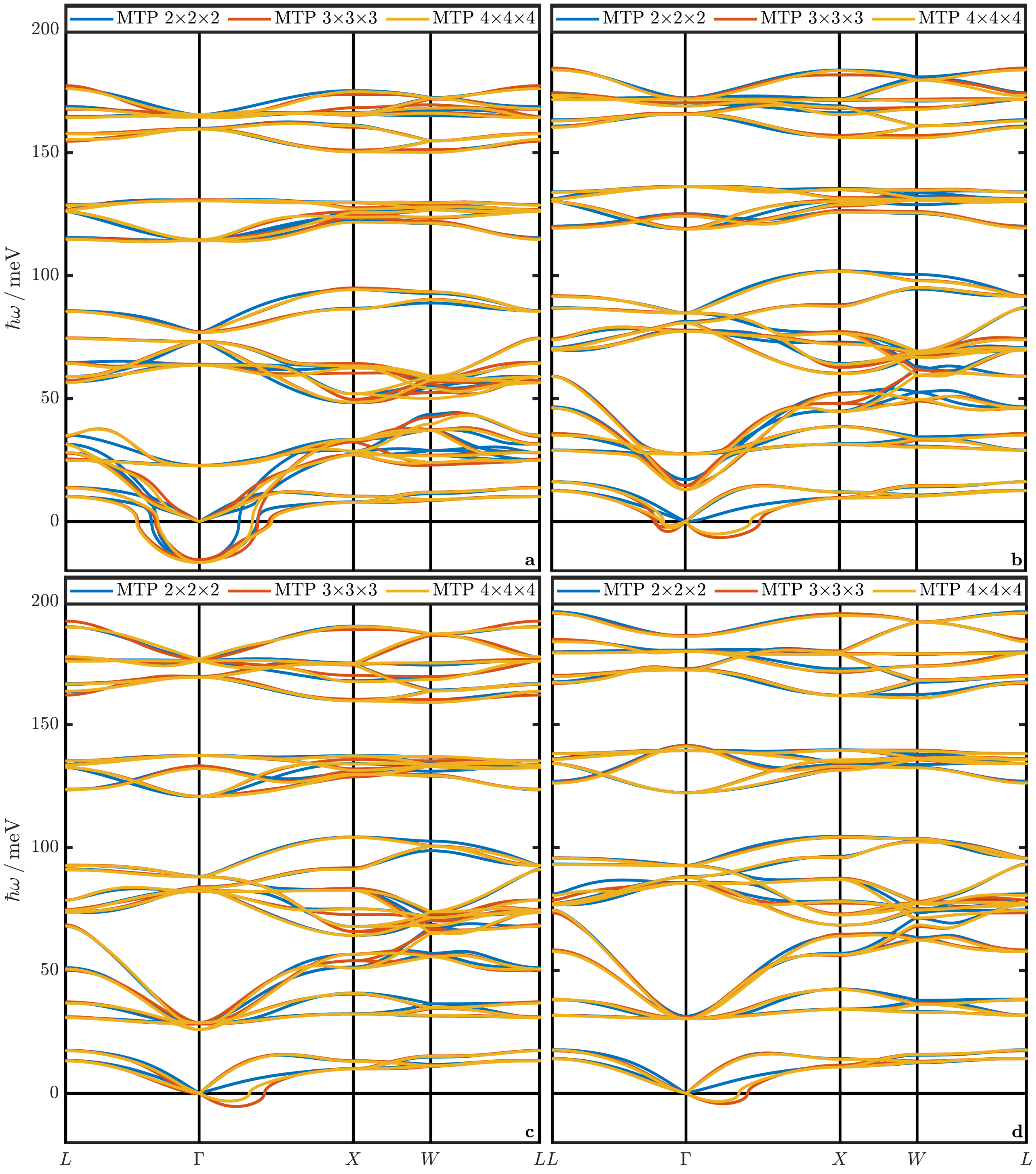

Supplementary Figure 5: **Convergence of the phonon dispersions with respect to supercell size:** SSCHA phonon dispersions including third-order terms for $n \times n \times n$ supercells with $n = 2, 3, 4$ (blue, red, and ochre lines respectively) obtained using MTPs. **a** 10 GPa, **b** 20 GPa,**c** 25 GPa, and **d** 30 GPa. The imaginary frequencies close to Γ on the path $\Gamma - X$ for $n = 3, 4$ in **c** and **d**, and additionally on $L - \Gamma$ in **b** are an artifact of the interpolation. All frequencies at **q** vectors commensurate with the corresponding supercell are positive. The imaginary region becomes smaller with increasing n, which suggests that this issue can be solved by using even higher supercells. However, the needed computational cost in terms of time and memory goes beyond the scope of this work.

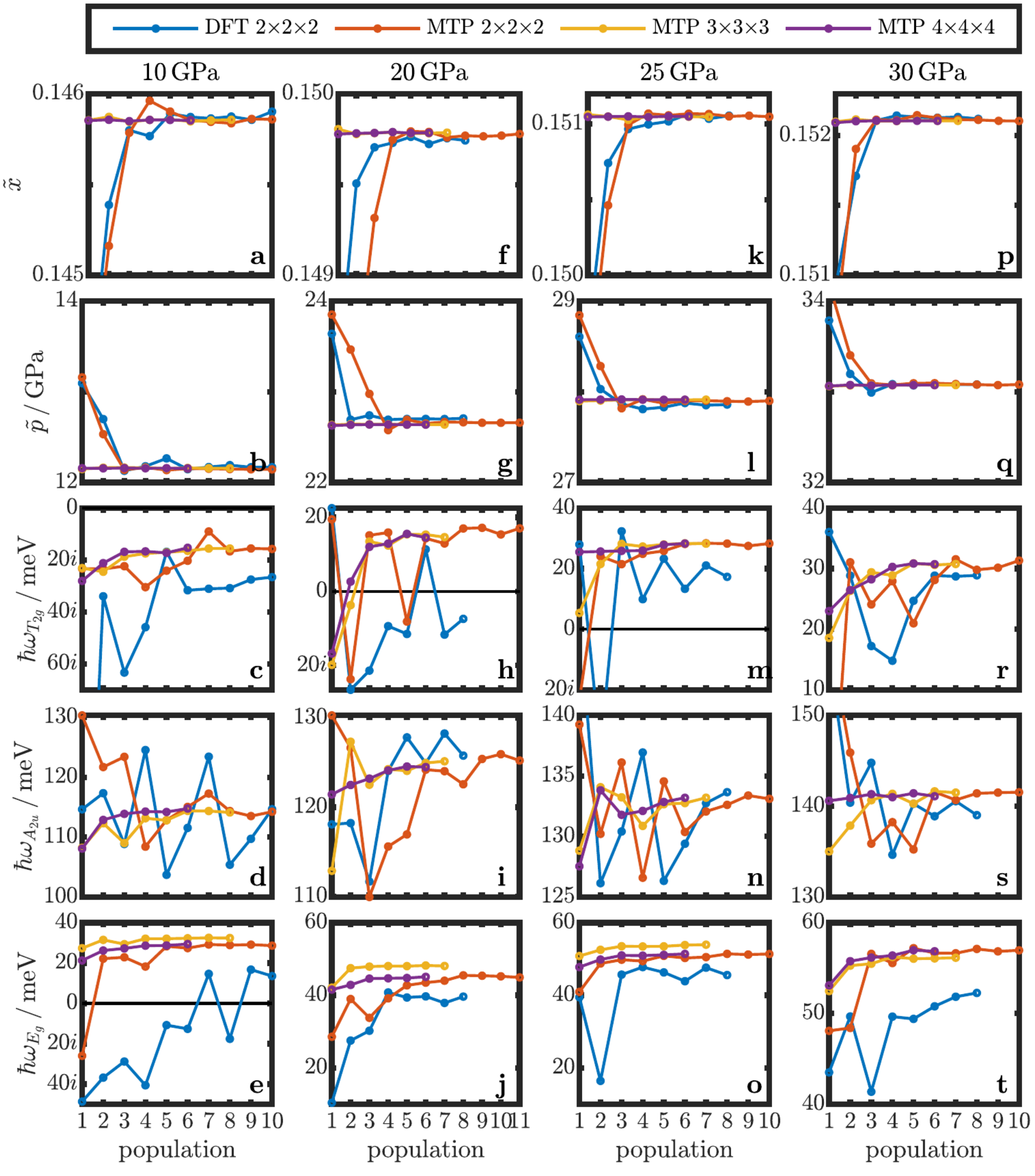

Supplementary Figure 6: **Convergence of structural parameters and physical phonon frequencies: a-e** $\tilde{x}$, $\tilde{p}$, and phonon frequencies for the T_{2g} and A_{2u} mode at Γ, and for the E_g mode at X at 10 GPa, **f-j** at 20 GPa, **k-o** at 25 GPa, **p-t** at 30 GPa. The plotted quantities are obtained at the end of the SSCHA minimization for each population. The phonon frequencies are obtained from the positional free energy Hessian including the third order terms. The calculations are done in $n \times n \times n$ supercells (DFT results for $n = 2$ in blue, MTP results for $n = 2, 3, 4$ in red, ochre and purple, respectively.)

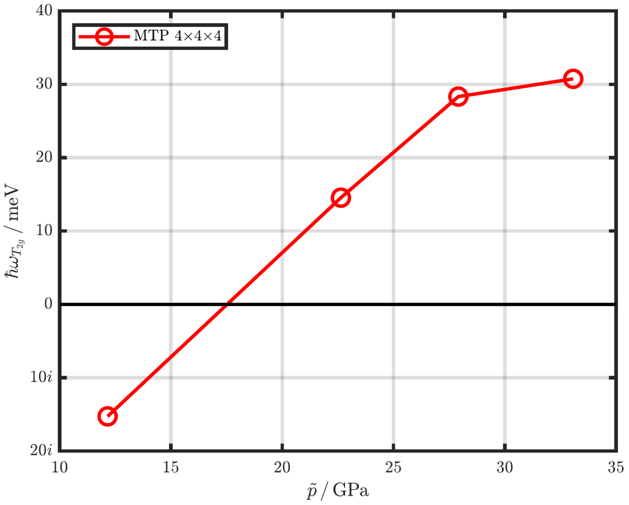

Supplementary Figure 7: **Dynamical (in)stability as a function of pressure:** SSCHA phonon frequency of the T_{2g} mode at Γ over SSCHA pressure $\tilde{p}$. The values correspond to the 4×4×4 supercell calculation with MTPs.

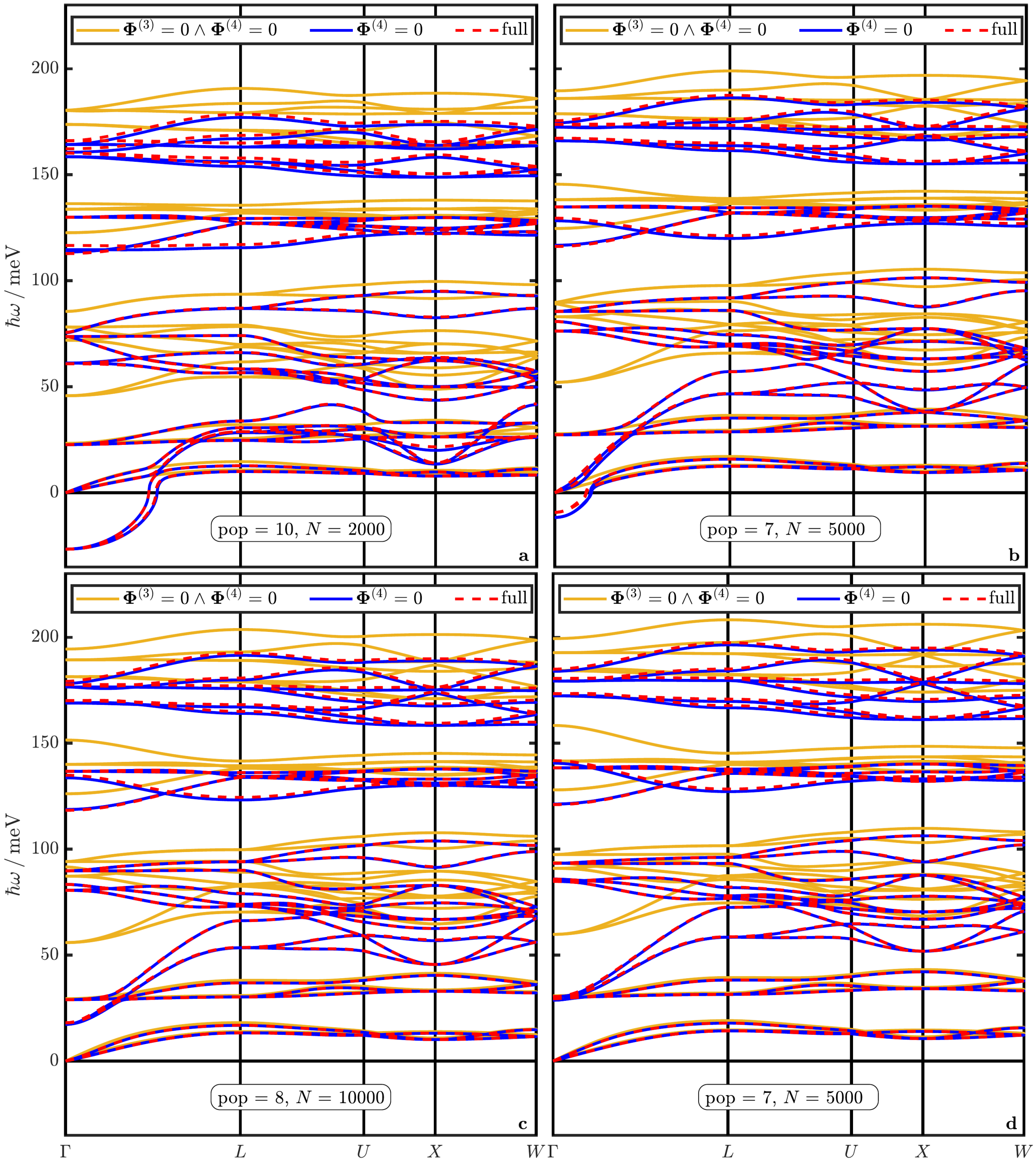

Supplementary Figure 8: **SSCHA phonon dispersions in different levels of approximation:** SSCHA phonon dispersions at **a** 10 GPa, **b** 20 GPa, **c** 25 GPa, and **d** 30 GPa including only the second order ($\Phi^{(2)}$, solid ochre lines), second and third second order terms ($\Phi^{(3)}$, solid blue lines), or all terms (dashed red line) in the positional free energy Hessian in Supplementary eq. (6). $\Phi^{(2)}$ correspond to the SSCHA auxiliary frequencies. The inclusion of the fourth order terms $\Phi^{(4)}$ (dashed red line) introduces only minor changes in the phonon frequencies compared to the inclusion of the third order terms (solid blue line), with maximum differences in higher modes in the order of 1 meV. The shown dispersions are obtained in 2×2×2 supercell within the SSCHA calculations with DFT for the population number (pop) and number of individuals (N) indicated in the panels.

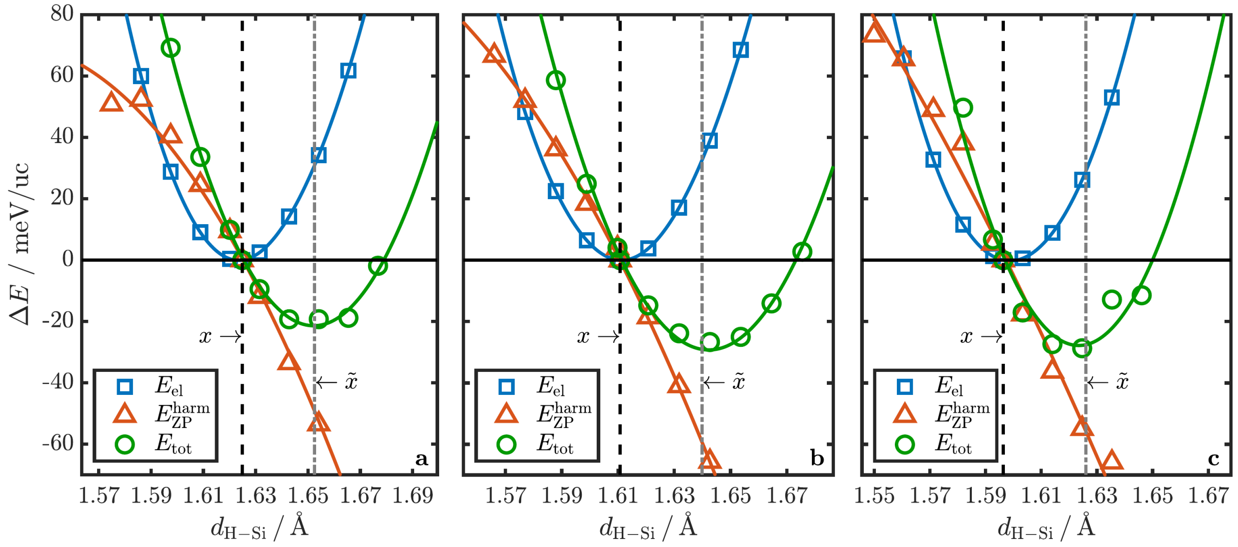

Supplementary Figure 9: **Electronic total energy, ZPE and resulting total energy** as a function of H-Si distance for **a** 10 GPa, **b** 20 GPa, and **c** 30 GPa. A detailed description and the data for 25 GPa can be found in Fig. 4 in the main text.

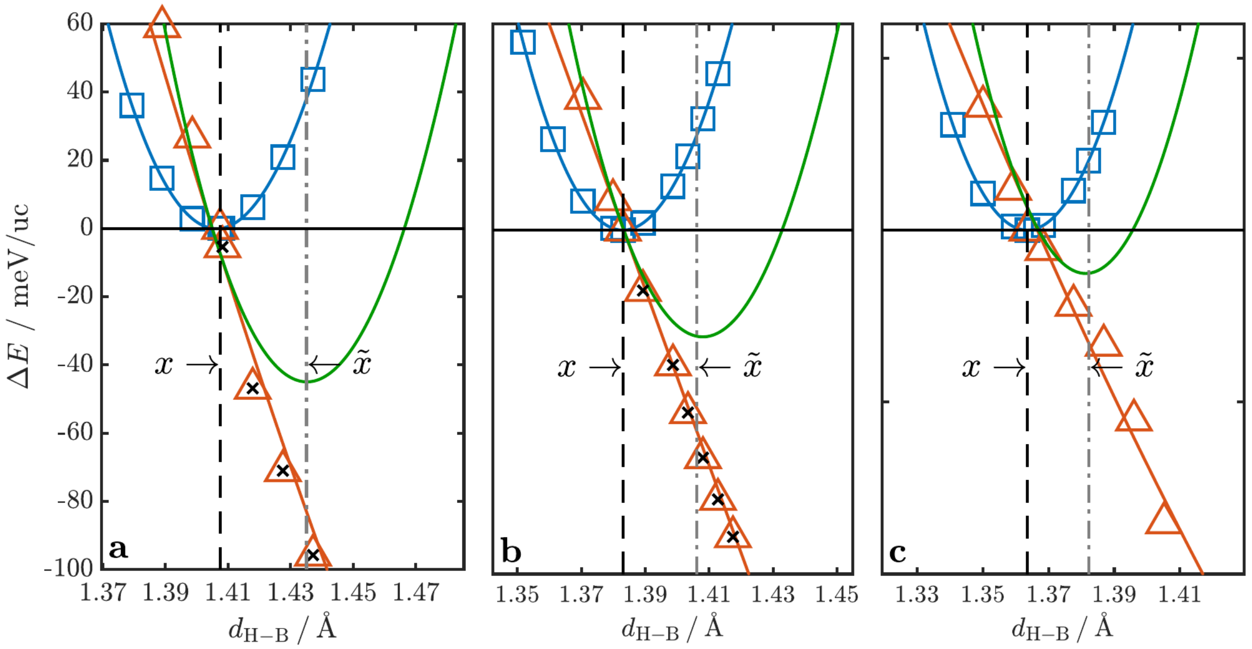

Supplementary Figure 10: **Electronic total energy, ZPE and total energy for $\mathbf{LaBH_8}$** as a function of H-B distance for **a** 50 GPa, **b** 75 GPa, and **c** 100 GPa. The black crosses mark calculations with imaginary harmonic phonon frequencies. Among these, we find a maximum of two non-degenerate modes per H-B distance in the BZ which we exclude in the ZPE calculation. The harmonic total energy minima in **a**, **b**, and **c** are at $d_{\text{H-B}} = 1.435$, 1.408 and 1.381 Å, respectively, which is in very good agreement with the values obtained within SSCHA and reported in Ref. [14].

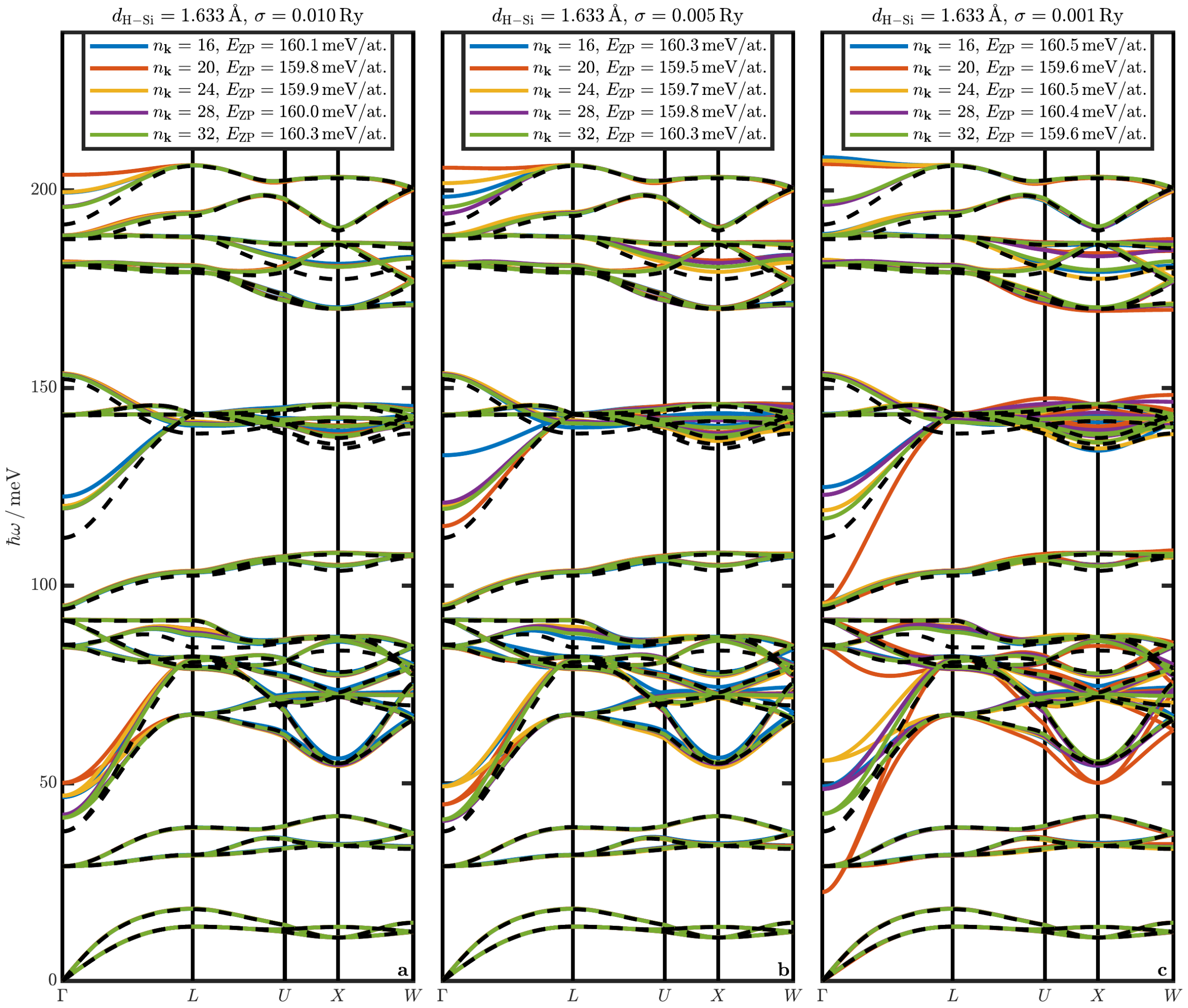

Supplementary Figure 11: **Phonon convergence in the total energy minimum structure:** Harmonic phonon dispersions for various **k**-grids and smearing values in $BaSiH_8$ at 25 GPa with a H-Si distance $d_{\text{H-Si}} = 1.633\,\text{Å}$, corresponding to the total energy minimum shown in Fig. 4 in the main text. The coloured solid lines represent different $n_{\mathbf{k}} \times n_{\mathbf{k}} \times n_{\mathbf{k}}$ **k**-grids, where $n_{\mathbf{k}} = 16, 20, 24, 28, 32$. The panels **a**, **b**, and **c** show the calculations for smearing values $\sigma = 0.010, 0.005$, and $0.001\,\mathrm{Ry}$. The zero-point energies E_{ZP} for each setting are indicated in the legend. The dashed lines represent the phonon dispersion corresponding to the settings used in the main text ($n_{\mathbf{k}} = 12$, $\sigma = 0.010\,\mathrm{Ry}$, $E_{\mathrm{ZP}} = 159.2\,\mathrm{meV/atom}$). Apart from some changes in three modes at Γ (T_{2g}, E_g, and A_{1g} which we already identified as particularly hard to converge and strongly non-parabolic), these convergence studies demonstrate that the phonon dispersions and zero-point energies in $BaSiH_8$ are in general very robust with respect to **k**-grid density and smearing parameter.

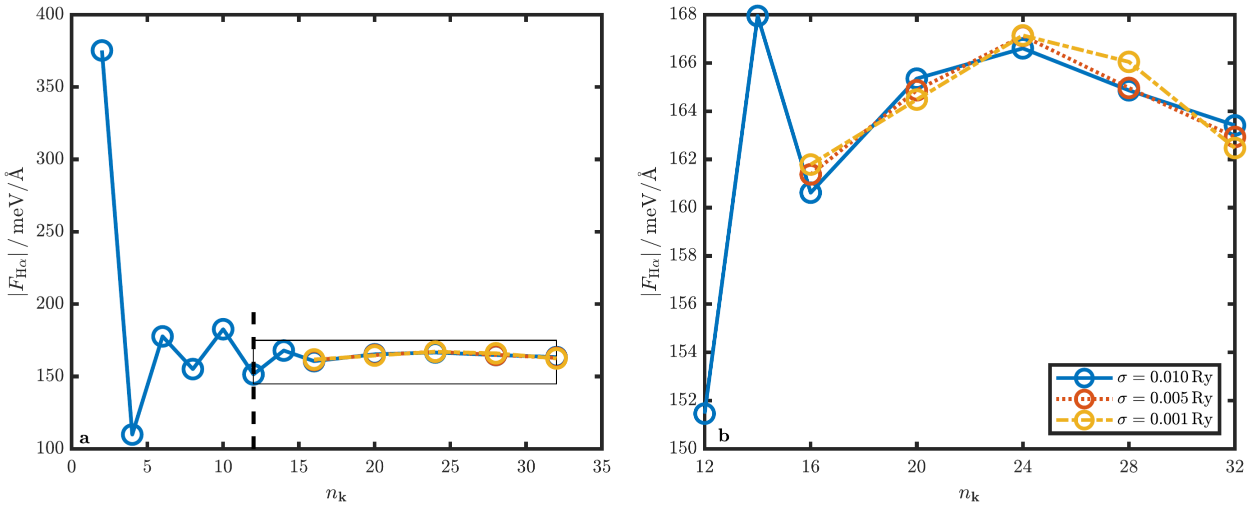

Supplementary Figure 12: **Force convergence in the total energy minimum structure:** Absolute value of the DFT force components on the H atoms $|F_{\mathrm{H}\alpha}|$ $(\alpha = x, y, z)$ for the different numerical settings described in Supplementary Fig. 11. **a** Overview including coarse grids down to $n_{\mathbf{k}} = 2$. The dashed line marks the computational settings in the main text. **b** Detail region indicated by the box in **a**.

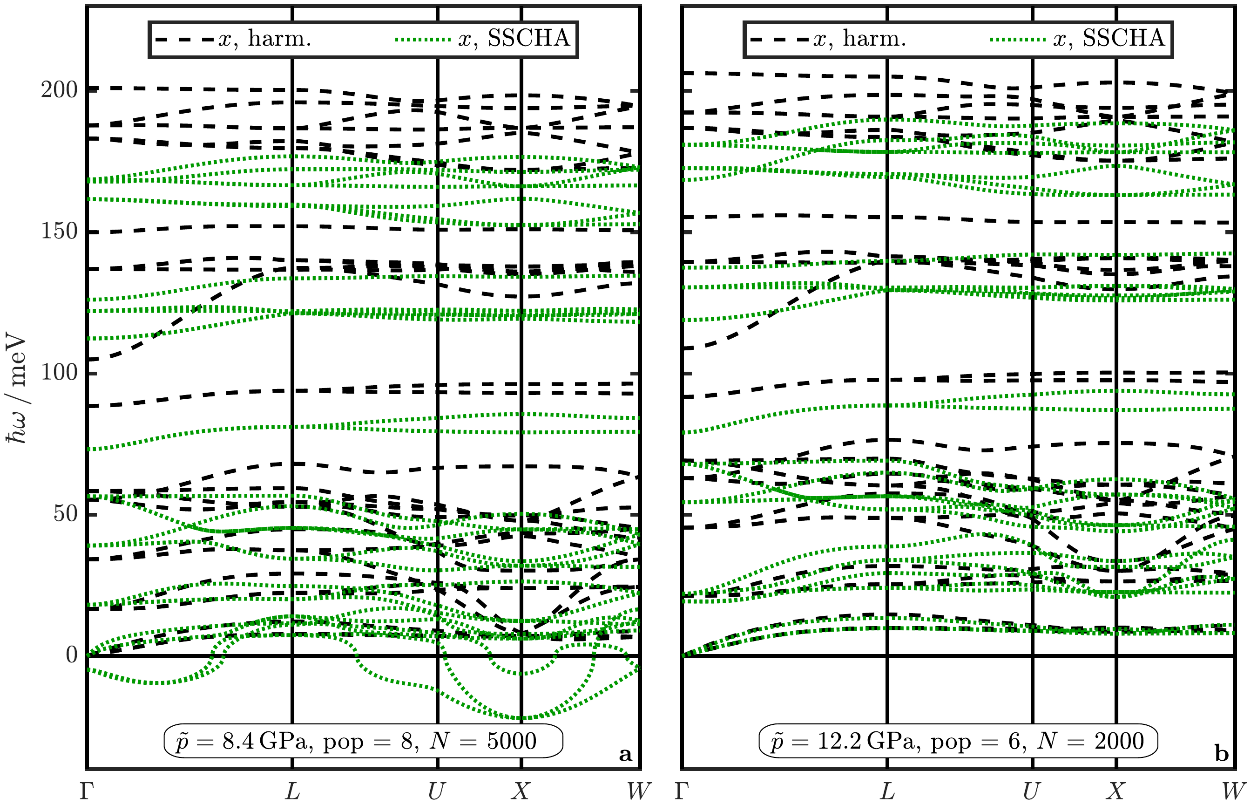

Supplementary Figure 13: **SSCHA phonon dispersions without relaxation** at **a** 5 GPa and **b** 10 GPa.The atomic positions are fixed to the DFT relaxed value x. The shown dispersions are obtained in 2×2×2 supercells within the SSCHA calculations with DFT for the population number (pop) and number of individuals (N) indicated in the panels. The SSCHA pressure is indicated by $\tilde{p}$.

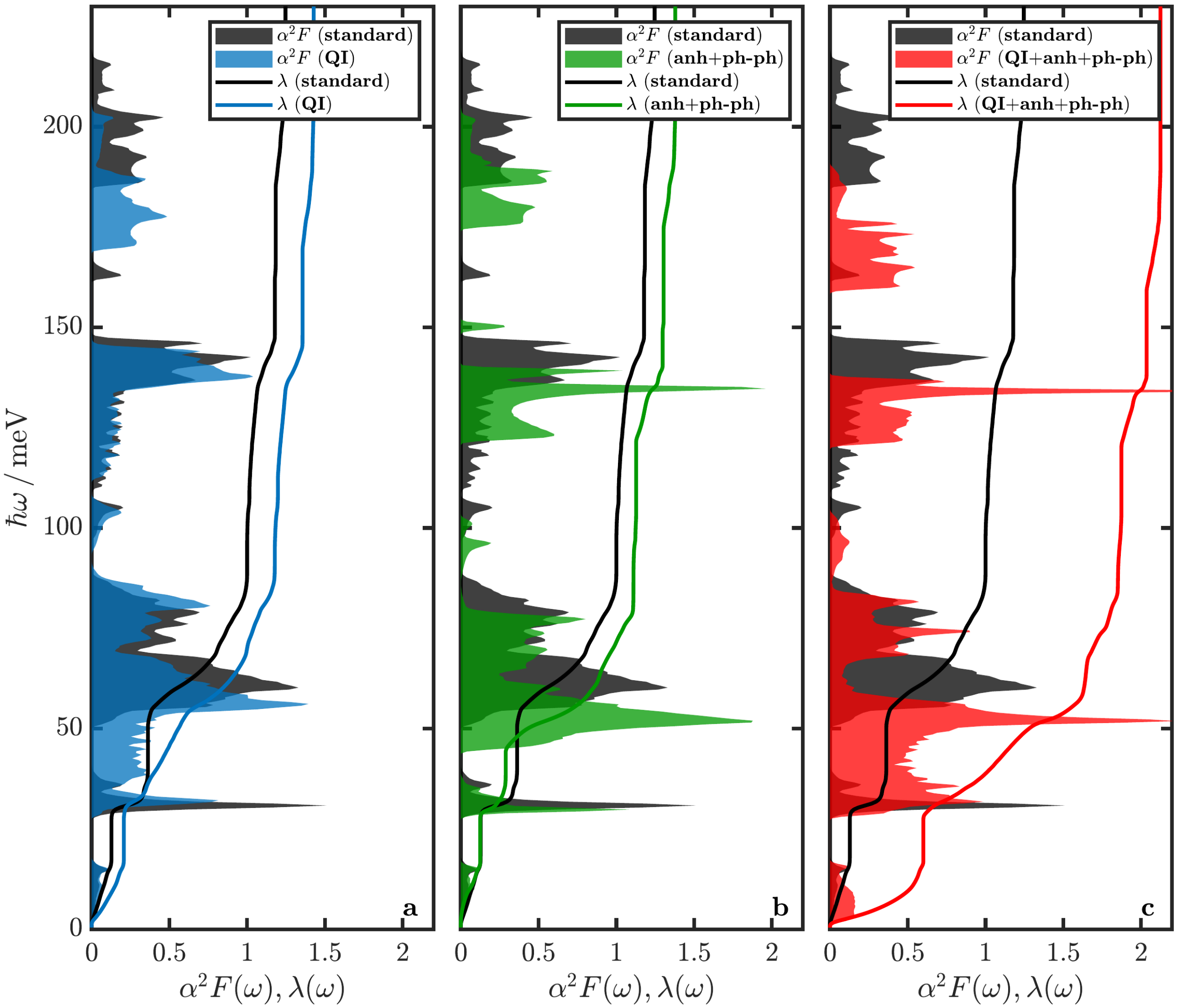

Supplementary Figure 14: **Eliashberg spectral functions:** Eliashberg spectral functions $\alpha^2F(\omega)$ (shaded areas) and cumulative *ep* coupling $\lambda(\omega)$ (solid lines) for the cases shown in Tab. III in the main text. The harmonic (**standard**) quantities are plotted in black as a reference in each panel.

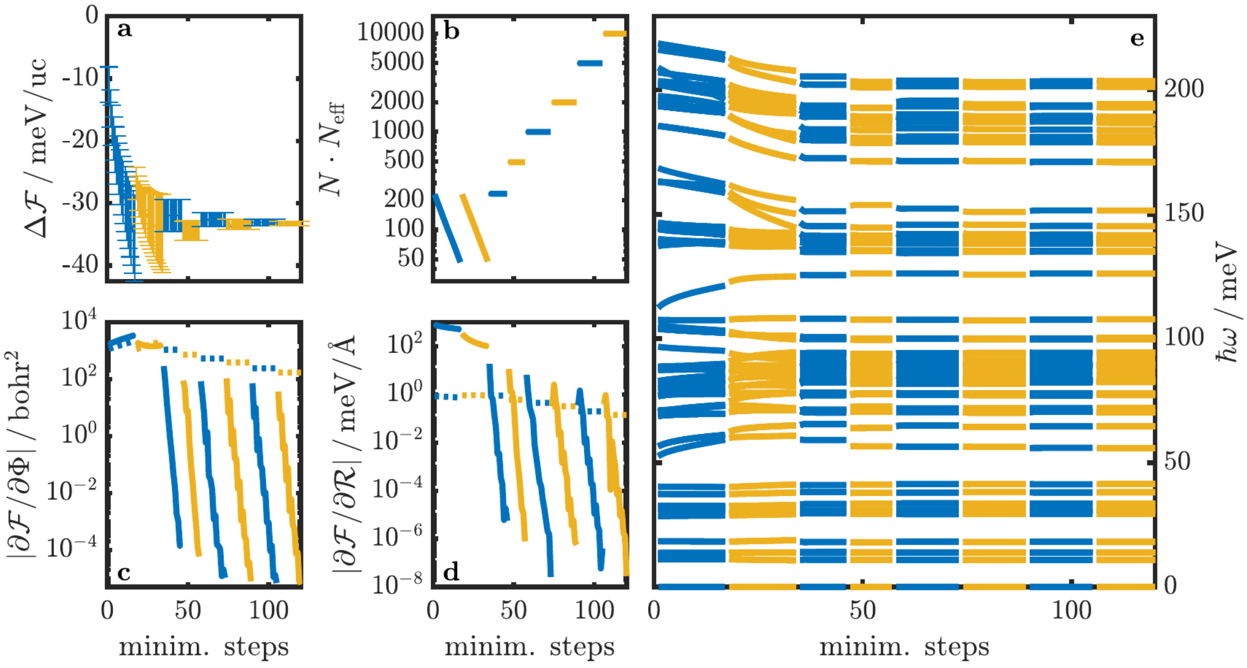

Supplementary Figure 15: **Convergence of the SSCHA minimization** in the 2×2×2 supercell with DFT calculations at 25 GPa with respect to minimization steps (alternating colours represent different populations): **a** SSCHA free energy $\mathcal{F}$ ± the stochastic error as errorbars, **b** Kong-Liu effective sample size N_{eff} times population size N, **c** modulus of the free energy gradient with respect to the auxiliary force constants (solid lines) and the corresponding stochastic error (dashed lines), **d** modulus of the free energy gradient with respect to the mean atomic positions $\mathcal{R}$ (SSCHA total force, solid lines) and the corresponding stochastic error (dashed lines), and **e** SSCHA auxiliary frequencies (eigenvalues of Φ).

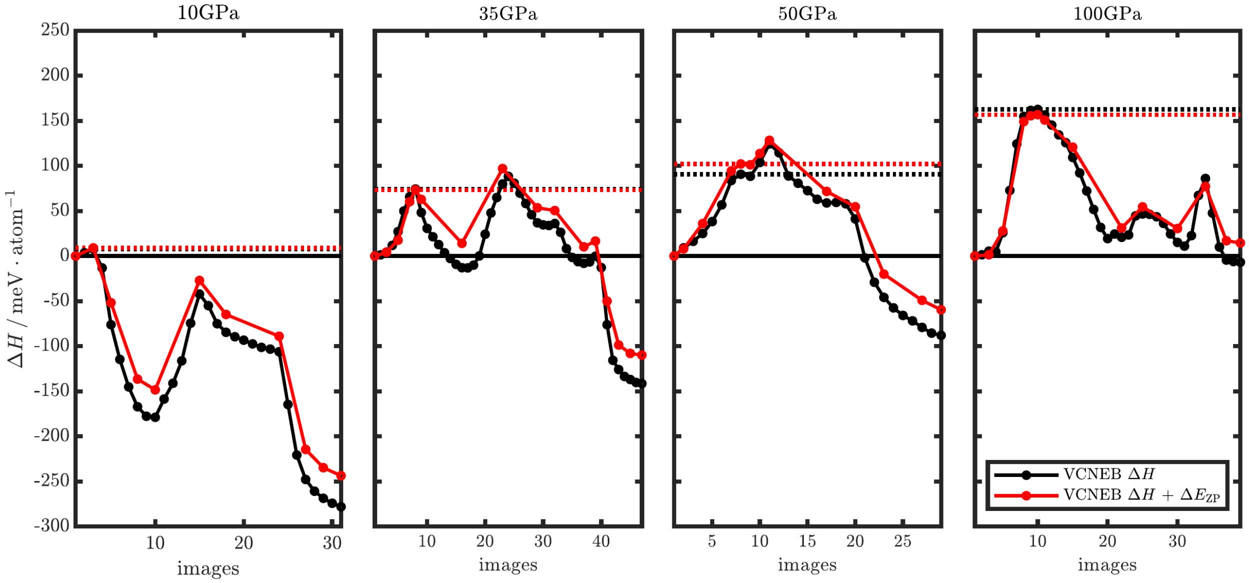

Supplementary Figure 16: **VCNEB calculations** showing the effects of including zero-point energies (ZPE) on the enthalpy barriers. The black lines show the calculations without ZPE (as reported in our previous work in Ref. [13]) and the red lines results of calculations where ZPE corrections have been included for selected structures. The dashed lines denote the determined barrier height. Note: As the intermediate structures within VCNEB may not always correspond to local minima of the potential energy surface imaginary phonon modes can appear, which, for a rough estimate of the size of ZPE, have been neglected.

SUPPLEMENTARY REFERENCES

[1] P. Giannozzi *et al.*, Advanced capabilities for materials modelling with Quantum ESPRESSO, J. Phys. Condens. Matter. **29**, 465901 (2017).

[2] D. R. Hamann, Optimized norm-conserving Vanderbilt pseudopotentials, Phys. Rev. B **88**, 085117 (2013).

[3] J. P. Perdew, K. Burke, and M. Ernzerhof, Generalized gradient approximation made simple, Phys. Rev. Lett. **77**, 3865 (1996).

[4] M. Born, Die Gültigkeitsgrenze der Theorie der idealen Kristalle und ihre Überwindung, in *Festschrift zur Feier des Zweihundertjährigen Bestehens der Akademie der Wissenschaften in Göttingen: I. Mathematisch-Physikalische Klasse* (Springer Berlin Heidelberg, Berlin, Heidelberg, 1951) pp. 1–16.

[5] D. J. Hooton, LI. A new treatment of anharmonicity in lattice thermodynamics: I, The London, Edinburgh, and Dublin Philosophical Magazine and Journal of Science **46**, 422 (1955).

[6] T. R. Koehler, Theory of the Self-Consistent Harmonic Approximation with Application to Solid Neon, Phys. Rev. Lett. **17**, 89 (1966).

[7] N. R. Werthamer, Self-consistent phonon formulation of anharmonic lattice dynamics, Phys. Rev. B **1**, 572 (1970).

[8] L. Monacelli, R. Bianco, M. Cherubini, M. Calandra, I. Errea, and F. Mauri, The stochastic self-consistent harmonic approximation: calculating vibrational properties of materials with full quantum and anharmonic effects, Journal of Physics: Condensed Matter **33**, 363001 (2021).

[9] R. Bianco, I. Errea, L. Paulatto, M. Calandra, and F. Mauri, Second-order structural phase transitions, free energy curvature, and temperature-dependent anharmonic phonons in the self-consistent harmonic approximation: Theory and stochastic implementation, Phys. Rev. B **96**, 014111 (2017).

[10] L. Monacelli, I. Errea, M. Calandra, and F. Mauri, Pressure and stress tensor of complex anharmonic crystals within the stochastic self-consistent harmonic approximation, Phys. Rev. B **98**, 024106 (2018).

[11] E. R. Margine and F. Giustino, Anisotropic Migdal-Eliashberg theory using Wannier functions, Phys. Rev. B **87**, 024505 (2013).

[12] S. Poncé, E. Margine, C. Verdi, and F. Giustino, EPW: Electron–phonon coupling, transport and superconducting properties using maximally localized Wannier functions, Comput. Phys. Commun. **209**, 116 (2016).

[13] R. Lucrezi, S. Di Cataldo, W. von der Linden, L. Boeri, and C. Heil, In-silico synthesis of lowest-pressure high-T_c ternary superhydrides, npj Computational Materials **8**, 119 (2022).

[14] F. Belli and I. Errea, Impact of ionic quantum fluctuations on the thermodynamic stability and superconductivity of $LaBH_8$, Phys. Rev. B **106**, 134509 (2022).
